# Microecological-metabolic links along the brain-gut-muscle axis in Tuina analgesia from the perspectives of gut metagenomics and untargeted metabolomics

**DOI:** 10.3389/fmicb.2026.1957609

**Published:** 2026-10-23

**Authors:** Xinlu Zhang, Huan Wang, Jie Xing, Hailong Zhao, Yangshengjie Liu, Jundong Jiao

**Affiliations:** 1 Department of Tuina, Affiliated Hospital of Changchun University of Chinese Medicine, Changchun, China; 2 Department of Thyroid Breast Surgery, Affiliated Hospital of Changchun University of Traditional Chinese Medicine, Changchun, China; 3 Acupuncture and Tuina College, Changchun University of Chinese Medicine, Changchun, China; 4 Department of Massage, Hangzhou Hospital of Traditional Chinese Medicine Affiliated to Zhejiang Chinese Medical University, Hangzhou, China; 5 Department of Tuina, Affiliated Hospital of Changchun University of Traditional Chinese Medicine, Changchun, China

**Keywords:** brain-gut-muscle axis, chronic pain, gut microbiome, metagenomics, microecological-metabolic links, multi-omics, Tuina, untargeted metabolomics

## Abstract

Chronic pain involves multilevel processes including central sensitization, peripheral tissue abnormalities, neuroimmune responses, metabolic alterations, and changes in gut microecology. Tuina and related manual therapies can influence local soft-tissue status, peripheral sensory input, autonomic activity, inflammatory responses, and muscle metabolism, whereas the links between their distal systemic effects and gut microecology remain insufficiently characterized. The brain, gut, and skeletal muscle can interact through autonomic pathways, immune-inflammatory signaling, circulating metabolites, myokines, and other distal signals, providing a biological context for analyzing cross-organ changes related to Tuina analgesia. A limited number of studies have reported changes in gut microbiota after Tuina, massage, or combined manual interventions; however, direct evidence continuously linking mechanical stimulation, microbial function, metabolic signaling, and pain improvement remains limited. Gut metagenomics extends analysis beyond species composition to strains, functional genes, and metabolic pathway potential, whereas untargeted metabolomics captures metabolic changes jointly shaped by the host and microbiota. Short-chain fatty acids, bile acids, tryptophan derivatives, and molecules related to lipid and energy metabolism have shown functional associations with neuroimmune regulation, sensory neural activity, and pain sensitization in different pain models. Myokines, metabolites, and extracellular vesicle signals derived from skeletal muscle also provide a possible biological basis for bidirectional gut-muscle communication. Nevertheless, most of these mechanisms originate from studies of pain, the microbiome, or exercise physiology and cannot be directly equated with mechanisms of Tuina analgesia. Integrated metagenomic and metabolomic analyses can identify candidate links involving microbial functions, metabolic alterations, host responses, and pain phenotypes, thereby narrowing the scope of subsequent functional validation. Their interpretation remains influenced by Tuina stimulation parameters, diet, gut transit, medication use, circadian rhythms, and other host factors. Standardization of the intervention, longitudinal multi-omics observation, targeted quantification, functional experiments, and cross-center replication will help clarify the temporal relationships and functional significance of these changes. Microecological-metabolic links across the brain, gut, and skeletal muscle can therefore serve as a cross-organ framework for investigating the systemic effects of Tuina analgesia, although specific pathways and their clinical predictive value still require direct experimental and prospective validation.

## 1 Introduction

Chronic pain involves multilevel alterations in peripheral nociceptive input, central sensitization, neuroimmune regulation, and psychosocial and behavioral factors, and its development and persistence are often accompanied by substantial interindividual variability (Cohen et al., 2021; Volcheck et al., 2023). Some patients also experience restricted movement, sleep disturbance, emotional symptoms, and reduced quality of life, giving long-standing pain increasingly cross-system characteristics. Pharmacological therapy remains an important component of chronic pain management, but the efficacy of nonsteroidal anti-inflammatory drugs, antidepressants, anticonvulsants, and opioids varies across populations and diseases, while long-term treatment also requires consideration of adverse effects, tolerance, opioid-related dependence, and other safety concerns (Dowell et al., 2022; Shi and Wu, 2023). Accordingly, nonpharmacological approaches that can be integrated with exercise rehabilitation, psychological interventions, and lifestyle management have attracted increasing attention.

Tuina is an important form of traditional manual therapy and is mainly used for musculoskeletal pain conditions such as chronic low back pain, neck and shoulder pain, osteoarthritis-related pain, and myofascial pain. Randomized controlled studies and systematic reviews indicate that Tuina can reduce pain and improve function in some patients with chronic nonspecific low back pain, although marked differences exist among studies in indications, manipulation techniques, stimulation dose, control conditions, and study quality (Yang J. et al., 2025; Yang et al., 2023). Manual stimulation can induce multilevel biological responses involving local tissues, neural signaling, neuroimmune, neurovascular, neuromuscular, and neuroendocrine processes, but the extent to which these changes mediate clinical analgesia remains incompletely defined (Keter et al., 2025). Improvements in local soft-tissue status, mechanosensory input, autonomic regulation, and inflammatory responses may explain some immediate or short-term effects, whereas persistent analgesia and interindividual differences in treatment response remain less completely understood.

In recent years, links between gut microecology and chronic pain have received increasing attention. Systematic reviews and meta-analyses have identified changes in gut microbial composition and diversity in some chronic pain populations, but pain type, geography, diet, medication exposure, and analytical methods all affect consistency across studies, and no stable, broadly generalizable pain-associated microbial signature has yet emerged (Goudman et al., 2024). Gut microbiota and their metabolites may participate in pain regulation through the intestinal barrier, mucosal immunity, circulating inflammatory signals, vagal pathways, and autonomic activity (Ho et al., 2025). At the same time, chronic musculoskeletal pain is often accompanied by local skeletal-muscle ischemia and hypoxia, microcirculatory abnormalities, mitochondrial dysfunction, oxidative stress, and disturbed energy metabolism (Wu K. et al., 2024). The brain, gut, and skeletal muscle therefore form potential cross-organ connections in which neural, immune, endocrine, and metabolic signals may jointly transmit information. The brain-gut-muscle axis provides an integrative framework for these otherwise dispersed biological observations, but the complete causal chain in Tuina analgesia remains to be experimentally established.

Advances in metagenomics and metabolomics have further expanded this research direction. Metagenomics can characterize microbial communities at the species and strain levels while providing information on functional genes and metabolic pathway potential, whereas metabolomics captures metabolic alterations jointly shaped by the host and microbiota. Their combined use facilitates analysis of links among microbial functions, metabolic signals, and host phenotypes (Yang S. Y. et al., 2025; Puig-Castellví et al., 2023). Candidate mediators that have attracted particular attention include short-chain fatty acids (SCFAs), bile acids, tryptophan derivatives, and other neuroactive metabolites. SCFAs participate in microbiota-gut-brain communication; bile acids and signaling through receptors such as FXR and TGR5 are associated with neuroinflammation and pain regulation; and serotonin, kynurenine, and indole derivatives generated through tryptophan metabolism can affect intestinal barrier integrity, immune homeostasis, and neural function (Dalile et al., 2019; Lin et al., 2026; Tuck et al., 2023). These findings provide a biological basis for microecological-metabolic signaling in pain regulation but do not demonstrate that the same mechanisms operate in Tuina analgesia.

Compared with this broader mechanistic literature, studies directly examining gut microecological changes after Tuina remain limited. In an animal study, Tuina was accompanied by changes in gut microbiota and protein expression in mice with experimental ulcerative colitis (Wang et al., 2022), but the study focused on intestinal inflammatory disease rather than chronic pain mechanisms. Gut microbial changes have also been observed in patients with knee osteoarthritis receiving electroacupuncture combined with Tuina (Guo et al., 2025); because both interventions were administered, the observed changes cannot be attributed to Tuina alone. In another animal study, massage improved skeletal muscle spasm and was accompanied by changes in energy metabolism and the metabolite profile (Sun et al., 2026), indicating that muscle itself may be an important metabolically responsive tissue after mechanical stimulation. Existing studies separately address gut microecology, host metabolism, and muscle responses, but few have continuously linked Tuina mechanical stimulation to changes in microbial function, specific metabolites, receptor or target-organ responses, and pain improvement. Autonomic regulation, muscle-derived cytokines and metabolites, circulating signals, changes in sleep and emotional state, activity level, and the treatment context may also interact with microecological changes to shape systemic responses after intervention.

Relevant literature for this narrative review was identified through searches of PubMed and the Web of Science Core Collection from database inception to June 2026. Search terms were combined around four main themes: Tuina and related manual interventions (“Tuina,” “Tui Na,” “massage,” and “manual therapy”); chronic pain and major musculoskeletal pain conditions; gut microbiota and metagenomics (“gut microbiota,” “gut microbiome,” “metagenomics,” and “microbial functional genes”); and metabolomic or cross-organ signaling (“metabolomics,” “microbial metabolites,” “short-chain fatty acids,” “bile acids,” “tryptophan,” “brain-gut-muscle axis,” and “myokines”). Reference lists of key articles were also screened to identify additional relevant publications. Peer-reviewed studies directly involving Tuina or related manual interventions, primary mechanistic studies relevant to microbiome–metabolite–pain interactions, and methodological or review articles relevant to multi-omics analysis were preferentially considered. The literature search was used to support a broad and critical narrative synthesis rather than a formal systematic review or meta-analysis.

Within this evidence base, microecological-metabolic mechanisms relevant to Tuina analgesia can be considered in relation to cross-organ brain-gut-muscle links in chronic pain, local and systemic responses to Tuina mechanical stimulation, changes in gut microbial function, and host-microbial co-metabolism. Clinical studies, animal experiments, omics associations, and functional studies support conclusions of different strengths; therefore, microbial taxa, metabolites, and receptor pathways should be interpreted in the context of specific study designs and their direct relevance to Tuina. Particular attention should be paid to which changes have actually been observed in Tuina studies, which are derived mainly from mechanistic research in chronic pain or microbiome science, and which links remain speculative. Within this framework, metagenomics and untargeted metabolomics can be used to identify biologically plausible microbial functions and metabolic nodes and to guide subsequent longitudinal studies, targeted validation, and functional intervention.

## 2 Cross-organ brain-gut-muscle links and microecological-metabolic regulation in chronic pain

### 2.1 Cross-organ signaling among the central nervous system, gut, and skeletal muscle

The central nervous system, gut, and skeletal muscle are connected through cross-organ signaling involving the autonomic nervous system, neuroendocrine pathways, immune-inflammatory responses, circulating metabolites, and peripheral sensory input. Studies of chronic pain indicate that the central nervous system can influence intestinal motility, barrier function, and mucosal immune status through autonomic pathways and the stress axis, whereas gut microbiota and their metabolites can affect neuroinflammation and pain processing through circulatory, immune, and vagal routes (Ho et al., 2025; Magni et al., 2023). These interactions may contribute to central sensitization and pain persistence, although the microbial taxa, metabolites, and neural pathways involved vary substantially across pain conditions.

Skeletal muscle is an important peripheral source of signals in chronic musculoskeletal pain. Abnormal mechanical loading, local ischemia, impaired energy metabolism, oxidative stress, and inflammation can increase the excitability of muscle nociceptors and generate persistent peripheral input that contributes to central pain sensitization (Lam et al., 2024). At the same time, gut-derived metabolic and inflammatory signals may influence muscle energy metabolism, mitochondrial function, and immune status, while skeletal muscle itself has endocrine and metabolic signaling functions and can participate in distal organ regulation through myokines and other circulating signals (Yin et al., 2022; Saponaro et al., 2024). Evidence for signaling from muscle to the gut derives mainly from exercise and metabolic research, and its role in chronic pain and Tuina interventions remains to be established.

The brain, gut, and skeletal muscle can therefore be viewed as a cross-organ regulatory network that changes dynamically with disease state. Potential neural, immune, and metabolic interfaces connect central sensitization, gut microecological changes, and skeletal muscle metabolic abnormalities, but the evidence supporting individual links is uneven. In particular, mechanistic studies continuously connecting muscle-derived signals, changes in gut microecology, and pain phenotypes remain scarce (Xu and He, 2025). This framework provides a basis for examining the relationships among central sensitization, gut microbiota and their metabolites, and skeletal muscle abnormalities in the following sections.

### 2.2 Central sensitization and neuroimmune regulation

Central sensitization is an important neurobiological basis for persistent and amplified pain, characterized primarily by increased excitability and synaptic efficacy in central nociceptive pathways and clinically associated with hyperalgesia, allodynia, and enhanced temporal summation (Woolf, 2011). It can be initiated by sustained peripheral nociceptive input and is further influenced by inflammatory states and changes in central regulatory networks. The extent and dominant mechanisms of central sensitization differ among chronic pain conditions, and its biological significance therefore needs to be interpreted in relation to the specific pain phenotype (Kosek, 2024).

Neuroimmune responses contribute to the development and maintenance of central sensitization. Persistent peripheral stimulation and inflammatory signals can alter microglial and astrocytic states, promote the release of cytokines, chemokines, reactive oxygen species, and other mediators, and thereby modulate neuronal excitability and synaptic plasticity (Atta et al., 2023; Cheng et al., 2022). Sustained interactions between glial cells and neurons thus represent an important component of pain amplification and neuroplastic change, although their contribution varies with pain type, disease stage, and the source of peripheral stimulation.

Within the cross-organ brain-gut-muscle framework, central sensitization also serves as an important interface through which peripheral signals converge on pain-regulatory networks. Skeletal muscle ischemia, metabolic disturbance, and inflammation can produce sustained nociceptive input, whereas gut dysbiosis and associated metabolic and inflammatory signals may influence central neuroimmune states through circulatory, immune, vagal, and neuroendocrine pathways (Ho et al., 2025; Lam et al., 2024). Current links between gut microecological changes and central sensitization are derived mainly from chronic pain and brain-gut axis research; extending these findings to Tuina analgesia requires continuous temporal associations and functional validation. Central sensitization can therefore be regarded as a central interface linking gut-derived signals, sustained skeletal muscle input, and chronic pain.

### 2.3 Gut microecological dysbiosis and pain regulation

Changes in gut microecology have been observed in different chronic pain populations, but no stable microbial signature has yet been reproduced consistently across diseases. Systematic reviews and meta-analyses indicate alterations in microbial diversity and the abundance of selected taxa in some chronic pain populations, with limited consistency among studies (Goudman et al., 2024). Pain type, geography, dietary pattern, medication exposure, age, sample collection, sequencing, and statistical methods can all affect microbiome results, making single genera or diversity indices insufficient to represent the overall significance of microecological changes in chronic pain. At present, greater functional relevance may lie in determining whether microbial capacities related to SCFA production, bile acid transformation, tryptophan metabolism, mucosal barrier maintenance, and inflammatory regulation change in parallel with pain phenotypes.

Links between gut microecology and pain may involve barrier integrity, mucosal and systemic immunity, and neural signaling. Disruption of the intestinal barrier can increase the passage of microbial components and inflammatory mediators into the circulation and thereby affect systemic inflammation, while microbiota and their metabolites may act on peripheral and central pain networks through immune cells, enteroendocrine signaling, and vagal pathways (Di Vincenzo et al., 2024; Zhao et al., 2025). Some animal studies provide stronger functional support. For example, microbiota altered after resolution of inflammation can transfer a visceral hypersensitivity phenotype to germ-free recipient animals through fecal microbiota transplantation, indicating that specific microecological states can participate in pain persistence (Arzamendi et al., 2024). Such findings arise from specific diseases and animal models, and their generalizability remains constrained by model characteristics, sex, inflammatory background, and host status. Direct studies are still lacking on whether microbiota changes after Tuina show stable temporal and functional relationships with pain improvement; continuous validation integrating microbial function, metabolite changes, and pain phenotypes is therefore required.

### 2.4 Gut-derived metabolites and neuroimmune pain regulation

Compared with microbial composition, metabolites produced by microorganisms or jointly formed by the host and microbiota are closer to the functional layer of cross-organ signaling. SCFAs, bile acids, and tryptophan derivatives are among the candidate mediators most frequently examined in pain research and can influence host responses through G protein-coupled receptors, nuclear receptors, immune regulation, and neural afferent pathways (Zeng et al., 2025). SCFAs in particular are broadly linked to intestinal barrier function, immune homeostasis, and neural regulation. Recent research in neuropathic pain provides a more specific mechanistic example: butyrate produced by *Roseburia intestinalis* can activate GPR41-related vagal signaling and reduce pain hypersensitivity through a brainstem-to-amygdala neural circuit (Jiang et al., 2025). This finding connects a defined bacterial strain, metabolite, receptor, and neural circuit, providing relatively complete experimental support for microbial metabolic signaling in pain regulation.

Bile acids also have signaling functions beyond digestion. Animal experiments show that activation of TGR5 and FXR in the spinal dorsal horn can reduce mechanical hypersensitivity after nerve injury (Wu et al., 2023). More recent work indicates that hyodeoxycholic acid can ameliorate neuropathic pain through FXR-related signaling, accompanied by changes in intestinal barrier and inflammatory status (Lin et al., 2026). Tryptophan metabolism encompasses serotonin, kynurenine, and microbiota-derived indole molecules. Indole can act as an AhR ligand in host-microbial signaling (Hubbard et al., 2015), and population studies have also associated several tryptophan metabolites with the severity of osteoarthritis-related pain. However, these findings are predominantly observational and are insufficient to establish a causal role for specific tryptophan metabolites in chronic pain (Meléndez-Oliva et al., 2025).

The biological effects of these metabolic signals are strongly dependent on disease and tissue context. Metabolite concentration, receptor distribution, host immune status, sex, and the local microenvironment can all modify their effects; therefore, an increase in a microbial taxon or a metabolite does not necessarily correspond to a fixed analgesic or pronociceptive direction. For example, in a post-inflammatory visceral pain model, changes in SCFA-producing bacteria were not consistently aligned with actual fecal SCFA concentrations, and the pain phenotype showed marked sex dependence (Arzamendi et al., 2024). Interpretation of gut-derived metabolites should therefore consider microbial function, measured metabolite levels, relevant receptors or target pathways, and pain phenotypes together. These specific mechanisms have been established mainly in pain and microbiome research, and there is currently no evidence that the same microbiota-metabolite-receptor chains directly mediate Tuina analgesia.

### 2.5 Skeletal muscle as a metabolic, inflammatory, and endocrine signaling node in chronic pain

Skeletal muscle and related soft tissues are both major sites affected in chronic musculoskeletal pain and sources of persistent peripheral nociceptive input. Abnormal local mechanical loading, changes in microcirculation and perfusion, ischemia and hypoxia, and inflammatory responses can disrupt muscle energy metabolism, impair ATP production, oxidative phosphorylation, and redox homeostasis, and promote lactate accumulation, local acidification, and reactive oxygen species generation, thereby increasing nociceptor excitability (Wu K. et al., 2024; Lam et al., 2024). Mitochondrial function and quality control may also participate in this process, because abnormalities in mitochondrial morphology, calcium homeostasis, biogenesis, and mitophagy can alter muscle energy supply and stress adaptation (Inferrera et al., 2024). Findings on muscle oxygenation, microcirculation, and mitochondrial abnormalities in conditions such as fibromyalgia remain somewhat inconsistent; these alterations are therefore better regarded as possible peripheral mechanisms in selected chronic musculoskeletal pain states rather than universal features of all chronic pain.

Skeletal muscle also has endocrine and immunoregulatory functions and can release myokines and multiple metabolic signals involved in communication with distant organs. IL-6 was among the first recognized myokines, and skeletal muscle contraction can release IL-6 into the circulation, where it participates in energy metabolism and immune regulation (Pedersen and Febbraio, 2008). The effects of muscle-derived IL-6 are strongly dependent on timing and physiological context; a transient increase after exercise or muscle contraction is biologically distinct from persistently elevated IL-6 in chronic inflammation. Irisin, generated by processing of FNDC5, is another representative muscle-derived signal involved in energy metabolism and cross-organ regulation (Boström et al., 2012). In addition to protein myokines, lactate, other muscle-derived metabolites, and lipid signals can enter the circulation and may influence systemic metabolic and immune states (Saponaro et al., 2024). These secretory and metabolic functions create a potential link between local tissue changes in chronic pain and systemic biological responses.

Communication between the gut and skeletal muscle is bidirectional. Gut microbiota and their metabolites may affect muscle status through inflammation, energy metabolism, insulin sensitivity, and mitochondrial function, while muscle activity and the myokines and metabolic signals it releases may in turn modify the intestinal immune and metabolic environment (Xu and He, 2025; Li et al., 2024). For example, exogenous irisin administration in an animal model of colitis was accompanied by reduced intestinal inflammation and changes in microbial composition, providing experimental evidence that a muscle-related signal can influence the intestinal microenvironment (Huangfu et al., 2021). However, that study did not demonstrate the same effect for endogenous skeletal-muscle-derived irisin and did not involve chronic pain. Continuous functional validation linking myokines, muscle-derived metabolites, gut microecological changes, and pain phenotypes is still lacking, and their contribution to systemic responses after Tuina mechanical stimulation remains uncertain.

### 2.6 Cross-organ microecological-metabolic links and the persistence of chronic pain

Central sensitization, changes in gut microecology, and skeletal muscle metabolic abnormalities can coexist in some chronic pain states and may be linked across organs through neural, immune, endocrine, and circulating metabolic signals (Ho et al., 2025; Yin et al., 2022). Gut microbiota and their metabolites may influence neuroimmune status and peripheral tissue metabolism; skeletal muscle inflammation, impaired energy metabolism, and persistent nociceptive input can also contribute to pain sensitization; and autonomic and stress responses may further regulate intestinal and peripheral tissue states (Lam et al., 2024; Wallrapp and Chiu, 2024). These interactions may contribute to the persistence and heterogeneity of some chronic pain conditions, but the dominant components and their relative contributions differ across pain types.

Population studies currently provide mainly associative evidence linking chronic pain with microbiota, metabolites, and inflammatory states, whereas animal and functional studies support the ability of selected microbial taxa and metabolites to alter pain sensitivity and neuroimmune responses. However, complete chains linking a defined microbial taxon, metabolite, receptor, target organ, and pain phenotype have rarely been validated continuously (Goudman et al., 2024; Metwaly et al., 2025). Diet, medication, sleep, activity level, and psychological status may simultaneously influence gut microecology and pain phenotypes, further complicating interpretation of cross-organ relationships. The brain-gut-muscle axis can therefore be used to organize biological links across different levels, while the directness and causal strength of individual components must be judged according to study design. For Tuina, a central question is whether neuroimmune responses, gut microecology, metabolic signals, and muscle status show temporally coherent changes after mechanical stimulation and whether those changes have stable functional relationships with pain improvement (Figure 1).

**Figure 1:**
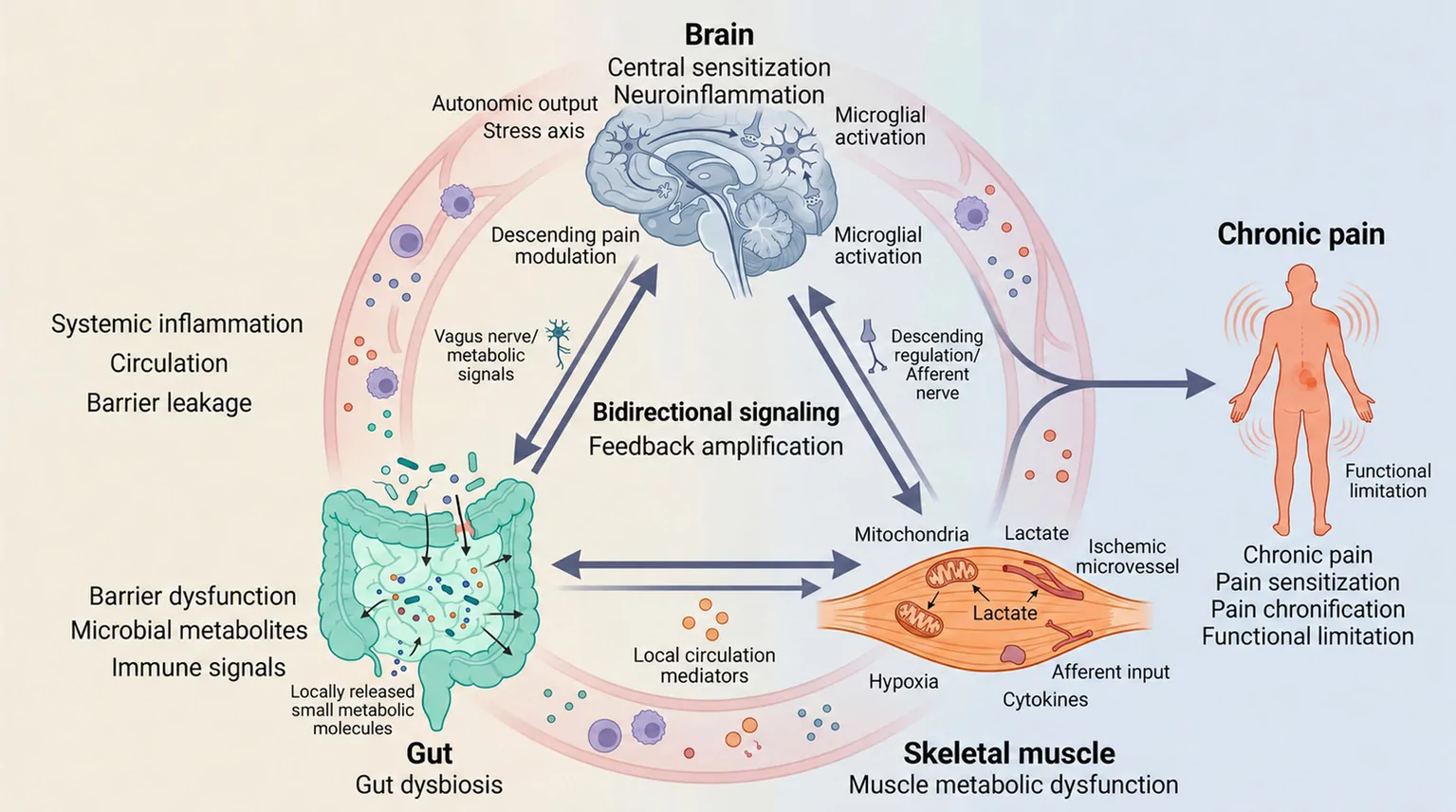
Overview of the pathological network of the brain-gut-muscle axis and microecological-metabolic imbalance mechanisms in chronic pain. The brain, gut, and skeletal muscle may communicate through autonomic signaling, circulating inflammatory mediators, microbial metabolites, and peripheral sensory input. Central sensitization, intestinal barrier and microbiota alterations, and muscle ischemia, mitochondrial dysfunction, and inflammation may jointly contribute to pain sensitization, chronification, and functional limitation. Several links remain to be directly validated in Tuina studies.

## 3 Clinical and biological basis of Tuina analgesia: from mechanical stimulation to systemic regulation

### 3.1 Clinical efficacy and research heterogeneity of Tuina for chronic musculoskeletal pain

Tuina and related manual therapies are used mainly for musculoskeletal pain conditions such as chronic low back pain, neck and shoulder pain, osteoarthritis-related pain, and myofascial pain. Existing randomized controlled studies and systematic reviews indicate that Tuina can reduce pain and improve function in some patients, although the magnitude and duration of benefit differ across diseases and studies (Yang et al., 2023; Zhu et al., 2024). Broader evidence on massage and manual therapy likewise suggests short-term improvement in some pain conditions, but most conclusions are supported by low- or very-low-certainty evidence, with little high-certainty evidence available (Mak et al., 2024). Clinical benefit should therefore be interpreted in relation to the specific pain condition, treatment protocol, and follow-up period rather than generalized across all patients with chronic pain.

Research heterogeneity is also a major limitation when interpreting the biological effects of Tuina. Trials differ substantially in manipulation type, stimulation intensity, treatment frequency and course, practitioner experience, control conditions, and concomitant therapies, while outcome measures and assessment time points are insufficiently standardized (Zhou et al., 2023). Even when similar clinical pain improvement is observed, different mechanical stimulation patterns may elicit different local tissue, neural, and systemic responses, making biomarkers and omics findings difficult to compare directly across studies. Mechanistic studies therefore require more precise recording and standardization of Tuina stimulation parameters and intervention exposure alongside clinical efficacy assessment, providing a consistent intervention basis for interpreting neuroimmune, gut microecological, and metabolic changes (Table 1). Table 1 summarizes chronic nonspecific low back pain, knee osteoarthritis, neck pain, myofascial pain syndrome, and fibromyalgia/widespread pain (Yang J. et al., 2025; Yang et al., 2023; Zhu et al., 2024; Gross et al., 2024; Feng et al., 2023; Makin et al., 2024; Chen et al., 2025; Carrasco-Vega et al., 2024; Bidonde et al., 2023).

**Table 1:** Clinical evidence for Tuina and related manual therapies in major chronic musculoskeletal pain conditions.

Pain condition/Reference	Intervention/evidence source	Main outcomes	Main findings	Major limitations
Chronic nonspecific low back pain (Yang J. et al., 2025; Yang et al., 2023)	Tuina; randomized trials and systematic review/meta-analysis	Pain intensity, disability (e.g., ODI), function, safety	Tuina has shown short-term improvements in pain and functional outcomes in some studies.	Heterogeneous techniques, controls and treatment courses; long-term durability and comparative effectiveness remain uncertain.
Knee osteoarthritis (Zhu et al., 2024; Feng et al., 2023)	Manual therapy/Tuina-related manipulation; systematic reviews and meta-analyses	Pain (VAS/WOMAC), physical function, safety	Manual therapy may reduce pain and improve function, particularly over short follow-up periods.	Variation in intervention type, treatment dose and study quality; evidence is not specific to Tuina in all included studies.
Neck pain (Gross et al., 2024; Makin et al., 2024)	Massage/manual therapy; systematic review/meta-analysis	Pain intensity, disability, adverse events	Massage/manual therapy may improve some pain and functional outcomes, but effects differ by comparator and follow-up.	Certainty is generally low; intervention definitions and protocols vary substantially.
Myofascial pain syndrome (Chen et al., 2025)	Manipulative therapy, massage, compression and related techniques; network meta-analysis	Pain intensity, pressure pain threshold, symptom relief	Several manipulative approaches show potential benefits for pain and pressure pain threshold.	Limited number of RCTs for individual techniques; substantial heterogeneity in technique and trigger-point localization.
Fibromyalgia/widespread pain (Carrasco-Vega et al., 2024; Bidonde et al., 2023)	Physiotherapy and nonpharmacological rehabilitation; manual therapy included within broader management	Pain, physical function, fatigue, sleep, quality of life	Nonpharmacological approaches can improve selected symptoms, but effects vary across intervention types.	Evidence is dominated by exercise/physiotherapy and multimodal care; direct Tuina-specific evidence is limited.

The table summarizes the main clinical evidence relevant to the manuscript scope. Manual therapy evidence is not automatically equivalent to Tuina-specific evidence.

### 3.2 Tuina manipulation parameters and biological effects

Tuina is highly operator-dependent, and technique type, treatment site, force, frequency, duration, contact area, and depth of tissue engagement together determine the actual characteristics of mechanical stimulation. Even when different studies use the same labels, such as “Tuina” or “massage,” their procedures and mechanical exposures may differ substantially, which can in turn affect the comparability of clinical and biological responses. Quantitative studies of Tuina manipulation show that parameters such as force waveform, peak force, mean force, and manipulation frequency can be recorded objectively with sensor-based devices. In Ding rolling manipulation, for example, force and frequency exhibit measurable operator-specific characteristics (Tune et al., 2023). These findings indicate that a technique name captures only part of the intervention, whereas the actual mechanical exposure is determined by multiple quantifiable parameters.

Different characteristics of mechanical stimulation may produce different tissue and neurophysiological responses. Studies of massage and related soft-tissue interventions show that variations in pressure delivery, tool characteristics, and stimulation patterns can affect deep-tissue compression, muscle activity, and selected neurophysiological measures (Kim et al., 2019; Kerautret et al., 2020). Mechanical deformation during Tuina may also alter local soft-tissue tension, mechanosensory input, and muscle state and may interact with autonomic responses. Because existing studies vary widely in intervention techniques, disease types, and outcome measures, a stable dose–response relationship has not been established. Differences in technique, stimulation intensity, rhythm, and depth change the actual mechanical exposure and may contribute to variation in local tissue, neural afferent, and systemic biological responses across studies.

Standardized reporting of Tuina interventions provides a basis for comparing biological effects across studies. STRICTOTM incorporates technical characteristics of the manipulation, treatment site, treatment duration and frequency, practitioner background, and control procedures into a reporting framework for Tuina/massage clinical trials (Zhang et al., 2023). In mechanistic studies, objective parameters such as pressure or applied force, rhythm, contact time, and procedural consistency can also be examined in parallel with local physiological measures, neuroimmune changes, and metabolic features. Advances in pressure sensing, kinematic recording, and local physiological monitoring are gradually shifting the description of Tuina mechanical stimulation from qualitative terminology toward quantifiable intervention exposure, providing a more stable basis for interpreting microecological and metabolomic changes across time points, individuals, and studies.

### 3.3 Local tissue and peripheral sensory responses to mechanical stimulation

Tuina acts first on the skin, fascia, muscle, and surrounding vascular and neural structures. Mechanical stimulation can deform soft tissues and may be accompanied by changes in tissue gliding, muscle tension, local perfusion, and inflammatory status. Fascia is richly innervated by sensory nerves and is involved in proprioception and nociception; studies of fascia and related manual interventions have also reported changes in tissue mechanics, local inflammation, and neuromuscular responses (Suarez-Rodriguez et al., 2022; Isaji et al., 2025). However, manipulation techniques, tissue sites, and measured outcomes differ considerably among studies, and the mediating relationship between local tissue changes and pain improvement remains incompletely defined.

Changes in local tissue status can further influence peripheral sensory input. In chronic musculoskeletal pain, inflammation, tissue stiffness, ischemia, and metabolic abnormalities may increase nociceptor sensitivity, allowing persistent peripheral input to contribute to pain sensitization; changes in tissue state and sensory input induced by mechanical stimulation may in turn modify this process (Langevin, 2021; Martínez-Pozas et al., 2023). Manual therapy studies indicate that pressure pain thresholds and other measures of pain sensitization can change after intervention, although the magnitude and duration of these effects are inconsistent. Local mechanical and sensory responses may therefore represent an initial interface through which Tuina influences higher-level neural regulation, with the specific effects depending on stimulation parameters, tissue condition, and pain phenotype.

### 3.4 Autonomic and neuroimmune regulation

Local mechanical and sensory input may be accompanied by broader neurophysiological responses. Studies of manual therapy have reported changes in neural, neurovascular, autonomic, and neuroimmune measures, although study quality and consistency vary substantially across mechanistic domains (Keter et al., 2025). Systematic reviews of autonomic effects indicate that some manual interventions can alter sympathetic- or parasympathetic-related indices, but the direction of effect depends on the intervention, measured variables, and study quality, and no stable intervention-specific autonomic response pattern has yet been established (Roura et al., 2021). Autonomic changes after Tuina may therefore contribute to pain regulation and systemic physiological responses, but a direct mediating relationship with analgesia remains uncertain.

Immune and endocrine changes provide another possible systemic link. Animal experiments show that massage-like stroking can alter T-cell numbers in the thymus and spleen and is accompanied by changes in noradrenergic innervation of lymphoid organs (Major et al., 2015). Massage studies in patients with fibromyalgia have also reported changes in pain, stress-related measures, and indices associated with cortisol rhythm (de Oliveira et al., 2018). These findings suggest that peripheral mechanical stimulation may be accompanied by coordinated neural, immune, and endocrine responses, but the evidence comes from different techniques and disease contexts and does not establish a unified pathway for Tuina analgesia. Autonomic, immune, and endocrine states also participate in the regulation of intestinal motility, barrier function, and mucosal immunity, providing a potential interface between systemic responses to mechanical stimulation and changes in the intestinal microenvironment (Macpherson et al., 2023). Overall, biological responses after Tuina mechanical stimulation can extend from local tissue and peripheral sensory changes to autonomic, immune, and endocrine levels, while the specific links among these components and their contributions to clinical analgesia remain to be clarified (Figure 2).

**Figure 2:**
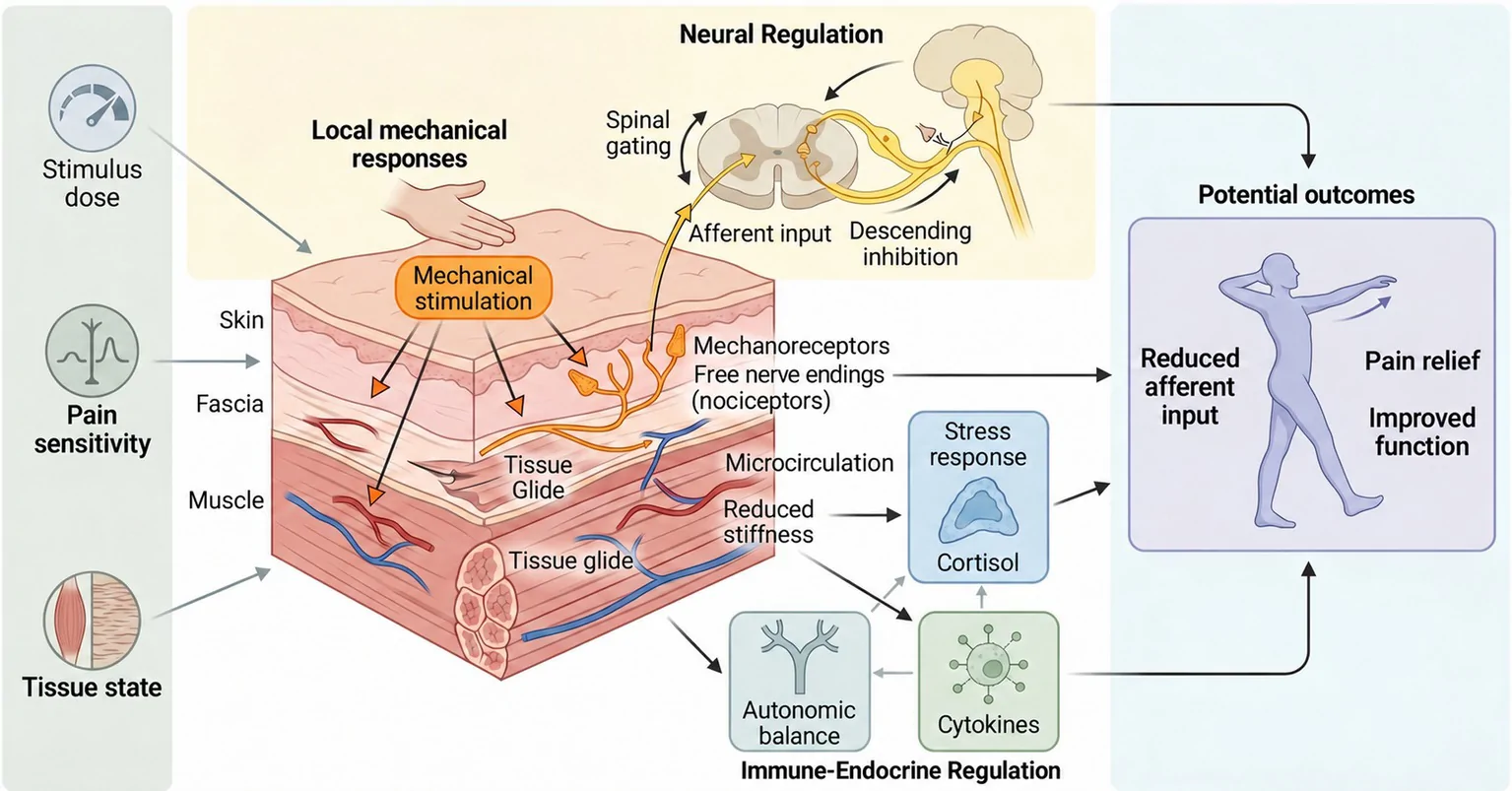
Local tissue responses and neuroendocrine-immune analgesic mechanisms induced by Tuina mechanical stimulation. Tuina may influence tissue gliding, muscle stiffness, local microcirculation, and mechanosensory and nociceptive input in the skin, fascia, and skeletal muscle. These responses may involve spinal and descending pain modulation, autonomic activity, inflammatory signaling, and stress-related endocrine responses, with potential effects on pain and function. Their magnitude may vary with manipulation parameters, tissue status, and pain phenotype.

### 3.5 From local biological effects to microecological-metabolic research

Mechanistic studies of Tuina and related manual therapies have documented multilevel changes in local tissues, pain sensitization, autonomic activity, inflammation, and endocrine responses, but these measures usually come from different studies and different time points, leaving limited continuity among them (Keter et al., 2025). Chronic pain itself is also characterized by variability in central sensitization, immune status, metabolic background, and gut microecology, making it difficult for a single local indicator to capture the systemic biological response to mechanical stimulation. Extending investigation from local tissue responses to cross-organ signaling therefore requires attention to intervention exposure, clinical phenotype, and temporal relationships across biological levels.

Gut metagenomics can further characterize microbial species and strain composition, functional genes, and metabolic pathway potential, whereas metabolomics reflects metabolic outputs jointly shaped by the host and microbiota. Combined analysis can link changes in microbial function to circulating or fecal metabolic signals (Yang S. Y. et al., 2025; Puig-Castellví et al., 2023). Such integration can be used to examine whether Tuina-related changes in microbial functions, SCFAs, bile acids, tryptophan derivatives, and host energy or lipid metabolism occur in parallel with neuroimmune states and pain phenotypes. Omics-level association networks alone cannot establish mediation and remain sensitive to sample size, confounding factors, batch effects, and statistical models (Metwaly et al., 2025; Eicher et al., 2020). The principal value of metagenomic and metabolomic data is therefore to identify candidate cross-level links and generate nodes that can be tested further by time-series analysis, targeted measurements, and functional validation.

## 4 Tuina-related gut microecological functional changes from a metagenomic perspective

### 4.1 Functional analytical value of gut metagenomics

16S rRNA sequencing can describe the composition and diversity of gut microbiota, but strain-level differences and microbial functions are inferred largely from taxonomic information. Methodological studies have shown clear limitations in the ability of 16S-based functional prediction to identify disease-related functional changes (Matchado et al., 2024). Metagenomic sequencing directly captures genes present within microbial communities and, beyond species- and strain-level resolution, enables analysis of functional genes, metabolic enzymes, and associated pathways. This provides a more direct basis for examining microbial functions related to carbohydrate utilization, SCFA production, amino acid metabolism, bile acid transformation, and inflammation (Yang S. Y. et al., 2025; Kim et al., 2024).

Gut microbiota studies in chronic pain show substantial heterogeneity across diseases and populations, and genus-level abundance changes are often poorly reproducible (Goudman et al., 2024). Integrating taxonomic changes with functional information can help determine whether different community structures nevertheless encode similar metabolic capacities. For example, functions related to SCFA production, bile acid transformation, tryptophan metabolism, and lipopolysaccharide biosynthesis may be contributed by multiple species, and their biological significance cannot be captured fully by increases or decreases in a single genus. Functional gene and pathway information from metagenomics therefore complements taxonomic composition and can be linked to subsequent metabolite measurements. In Tuina-related research, this layer of information may help determine whether intervention-associated microbiota changes are accompanied by changes in microbial functional potential and whether these changes are concordant with pain, inflammation, and host metabolic phenotypes.

### 4.2 Current evidence on Tuina-related changes in gut microbiota

Studies linking Tuina or related manual interventions with gut microbiota remain few and vary substantially in disease background and intervention type. In mice with experimental ulcerative colitis, Tuina was followed by changes in gut microbial composition together with alterations in colonic protein expression and related metabolic pathways (Wang et al., 2022). In rats with experimental insomnia, abdominal massage was also associated with changes in microbial structure, predicted functional pathways, and selected brain-gut peptides (Liu et al., 2025a). These findings indicate that manual stimulation can accompany changes in gut microecology and host biological state, but the relevant studies are concentrated in gastrointestinal disorders or non-pain models, and their relationship to analgesia in chronic pain remains unclear.

Pain-related evidence has so far come mainly from combined interventions. In patients with knee osteoarthritis receiving electroacupuncture plus Tuina, gut microbial structure and the abundance of selected taxa changed in parallel with improvements in pain and function (Guo et al., 2025). Because electroacupuncture was administered in both groups, the microecological changes cannot be attributed fully to Tuina. In addition, the study used 16S rRNA sequencing, so its findings were focused mainly on community composition and predicted functions rather than direct resolution of strains, functional genes, or actual metabolic outputs. Thus, Tuina-related studies have documented changes in microbial structure, but continuous data linking these changes to defined microbial functions, metabolites, and pain phenotypes are still lacking.

The effects of manual stimulation on gut microbiota may be related to changes in the host microenvironment. The enteric nervous system, autonomic activity, intestinal motility, mucosal secretion, barrier function, and local immune status jointly contribute to the maintenance of microbial niches (Sharkey and Mawe, 2023). Animal experiments further show that stress-related neural circuits can alter mucus secretion and gut microbial composition through duodenal glands, demonstrating that neural activity can influence microbial ecology by modifying the host tissue environment (Chang et al., 2024). As a peripheral mechanical stimulus, Tuina may create a similar indirect interface through autonomic and neuroimmune responses, but studies that simultaneously measure Tuina stimulation, the intestinal microenvironment, microbial function, and pain changes are not yet available. Current findings mainly indicate that Tuina and related manual interventions can coincide with changes in gut microecology; their specific role in analgesia requires further evaluation alongside functional genes, metabolites, and pain phenotypes.

### 4.3 Functional guilds of gut microbiota and pain regulation

The taxonomic composition of the gut microbiota does not map perfectly onto its functional activity. Different strains within the same genus or species can have distinct metabolic capacities, while similar metabolic functions can be performed by phylogenetically unrelated microorganisms. Functional guilds defined by co-abundance relationships and ecological functions can describe this pattern in terms of community cooperation and functional redundancy, providing another way to explain why taxonomic findings differ across studies even when functional changes are similar (Wu et al., 2021). This functional perspective is relevant to chronic pain because few genus-level features are consistently reproduced across diseases and populations, whereas microbiota-transfer studies indicate that changes in the microbial community as a whole can be accompanied by pain, immune, and metabolic phenotypes. For example, transplantation of microbiota from patients with fibromyalgia into germ-free mice can induce pain hypersensitivity together with immune activation and changes in metabolic profiles (Cai et al., 2025).

Microbial groups associated with SCFA production represent one of the more clearly characterized functional guilds in pain research. The capacity to produce butyrate is distributed across multiple members of the gut microbiota, including selected *Roseburia*, *Faecalibacterium*, and Lachnospiraceae taxa with substantial SCFA-producing potential. Mechanistically, *Roseburia intestinalis* has been shown to alleviate neuropathic pain by producing butyrate, which activates GPR41-related vagal signaling and modulates brainstem-to-amygdala neural circuits (Jiang et al., 2025). This study illustrates that a continuous link among microbial function, an actual metabolite, and a host receptor provides more biologically meaningful evidence for microbial involvement in pain regulation than a change in the abundance of a single genus alone.

Beyond SCFA production, bile acid transformation and tryptophan/indole metabolism also form functional modules relevant to pain regulation. Different intestinal microorganisms participate in deconjugation of primary bile acids and formation of secondary bile acids, whose products can influence inflammation, energy metabolism, and sensory nerve function through receptors including FXR and TGR5. In diabetic peripheral neuropathic pain, functional links have been reported among gut microbiota, bile acids, and TGR5/TRPV1 signaling (Chen et al., 2024). Tryptophan metabolism encompasses several branches involving kynurenine, serotonin, and microbiota-derived indoles, among which indole metabolites can participate in immune and neural regulation (Xue et al., 2023).

Microbial functions related to lipopolysaccharide synthesis, inflammation, and maintenance of the mucosal barrier may likewise contribute to pain-associated microenvironmental changes. Different functional guilds may cooperate or compete, and their net effects depend on community structure, functional gene composition, metabolite levels, and host state. Tuina-related studies currently remain focused largely on microbial composition and predicted function; metagenomic sequencing has not yet directly confirmed stable changes in these functional guilds after Tuina, nor is there functional validation showing that such changes occur in parallel with pain improvement. The functional characteristics of Tuina-associated microecological changes therefore remain to be defined.

### 4.4 Microbial functional genes and metabolic pathways

Metagenomics directly captures the gene composition of microbial communities, extending functional analysis from taxonomic inference to specific metabolic enzymes and pathways. Functions with particular relevance to pain and neuroimmune regulation include complex carbohydrate utilization, SCFA production, bile acid transformation, tryptophan metabolism, and inflammation-related microbial activities. Carbohydrate-active enzymes (CAZymes) participate in the degradation of dietary fiber and complex polysaccharides, generating substrates that can subsequently enter SCFA synthesis pathways. Terminal steps in butyrate production mainly involve functional genes such as butyryl-CoA:acetate CoA-transferase (but) and butyrate kinase (buk). Because different microbial members can generate butyrate through distinct pathways, functional gene abundance can complement taxonomic information when estimating butyrate-producing potential (Vital et al., 2013).

Bile acid metabolism likewise has a relatively well-defined microbial genetic basis. Bile salt hydrolase is encoded by bsh genes and catalyzes deconjugation of conjugated bile acids, generating substrates for subsequent transformations. Some intestinal bacteria also carry bile acid-inducible bai gene clusters that participate in 7alpha-dehydroxylation of primary bile acids and the formation of secondary bile acids (Larabi et al., 2023). Because bile acid derivatives can act on host receptors such as FXR and TGR5, changes in the abundance and composition of these genes may more closely reflect microbial functional status than changes in bile acid-associated genera alone. Tryptophan metabolism shows a similar pattern. For example, tnaA encodes tryptophanase, which converts tryptophan to indole, while other microorganisms can generate indole-lactic acid and related metabolites through enzymes including aromatic lactate dehydrogenases (Sinha et al., 2024). The same tryptophan substrate can therefore yield metabolites with different host effects depending on the organisms and enzyme systems involved.

In addition to these metabolic pathways, lipopolysaccharide biosynthesis and other functional modules related to mucosal inflammation, oxidative stress, and host–microbe interactions may contribute to the systemic inflammatory background associated with pain. Metagenomic data can be analyzed using resources and pipelines such as KEGG Orthology, MetaCyc, eggNOG, and HUMAnN to characterize functional genes and metabolic pathways and to identify the contribution of different species to specific functions (Yang S. Y. et al., 2025; Kim et al., 2024). Current Tuina-related microbiota studies have not directly demonstrated stable changes in but/buk, bsh/bai, tnaA, or inflammation-related functional modules. These genes are therefore better regarded as representative indicators for interpreting microbial functional changes rather than presumed molecular targets already involved in Tuina analgesia.

Metagenomics reflects the genetic and metabolic potential of microbial communities, and functional gene abundance is not equivalent to actual metabolic activity. Even when relevant metabolic genes are present, final metabolite output is influenced by substrate availability, microbial cross-feeding, intestinal pH, and the host environment. For example, the direction of microbial tryptophan metabolism can change substantially with dietary fiber availability and metabolic competition among community members (Sinha et al., 2024). Concordance among functional genes, fecal or circulating metabolites, and host phenotypes is therefore important when interpreting the biological meaning of microbial functions. Integrated metagenomic and metabolomic analysis can determine whether a change in a functional gene is accompanied by a corresponding change in metabolite levels and can provide more specific association nodes for subsequent receptor and pain-phenotype studies.

### 4.5 Confounding factors and statistical analysis in metagenomic research

The gut microbiome shows marked interindividual variability, and host physiology, lifestyle, and medication exposure can all alter microbial composition and function. In chronic pain populations, dietary pattern and recent food intake, gut transit time, bowel frequency, and stool consistency are particularly important because they directly influence the microbial growth environment and fecal microbiota measurements (Procházková et al., 2023; Vujkovic-Cvijin et al., 2020). Age, sex, BMI, comorbidities, physical activity, sleep status, and circadian rhythms can also contribute substantial variability. Sampling time itself can affect reproducibility of microbiome results, so fecal and blood samples collected at different times may incorporate additional circadian variation. Patients with chronic pain commonly use NSAIDs, opioids, and other analgesic or psychotropic medications; NSAIDs and opioids can alter gut microbiota, intestinal motility, and the mucosal environment, while antibiotics and probiotics can cause more direct microecological perturbations (Zádori et al., 2023). These factors may also correlate with pain severity, disease type, and treatment response, making pre- and post-intervention microbiome differences difficult to attribute fully to Tuina.

Microbiome data are compositional, sparse, and high-dimensional, and the statistical model selected can affect identification of differential taxa and functional pathways. MaAsLin2 can incorporate age, sex, BMI, diet, medication use, and other clinical variables into multivariable models to assess independent associations between microbial or functional features and clinical phenotypes, and it can accommodate cross-sectional and selected longitudinal designs (Mallick et al., 2021). For differential abundance analysis, ANCOM-BC addresses compositional microbiome data by correcting sampling-fraction bias, while ANCOM-BC2 extends this framework to multigroup comparisons, covariate adjustment, and repeated-measures settings (Lin and Peddada, 2020, 2024). In longitudinal datasets, repeated observations within the same participant can be handled with mixed-effects models including subject-specific random effects, while multiple testing across high-dimensional features should be controlled using false discovery rate procedures to reduce spurious discoveries.

Sample collection and laboratory workflows can also influence metagenomic results. Fecal collection time, preservation and freezing conditions, DNA extraction methods, sequencing platform and depth, host-read removal, taxonomic and functional annotation pipelines, and experimental batches can all introduce technical variation. Functional analyses are additionally affected by database coverage and algorithmic differences among KEGG, MetaCyc, eggNOG, HUMAnN, and related resources, so the same functional pathway may not yield identical results across pipelines. The STORMS checklist provides a structured reporting framework for human microbiome studies covering study design, sample handling, bioinformatics workflows, statistical analysis, and data reporting, thereby improving transparency and reproducibility across studies (Mirzayi et al., 2021). Even after extensive covariate adjustment and technical quality control, metagenomic results primarily describe associations between microbial features and clinical phenotypes; functional links involving metabolites, receptors, and pain effects require longitudinal and experimental validation.

## 5 Host-microbial co-metabolic changes related to Tuina from an untargeted metabolomic perspective

### 5.1 Metabolic profiling value of untargeted metabolomics

Untargeted metabolomics can detect a broad range of small-molecule metabolites in biological samples and can reflect host-microbial metabolic states across amino acids, lipids, organic acids, bile acids, and intermediates of energy metabolism. Compared with targeted approaches that predefine analytes of interest, untargeted strategies are better suited to discovering previously unrecognized metabolic changes and candidate pathways and therefore have substantial exploratory value in mechanistically heterogeneous conditions such as chronic pain (Teckchandani et al., 2021; Aroke and Powell-Roach, 2020). Existing metabolomic studies of chronic pain have reported changes in amino acid, lipid, energy-metabolism, and oxidative-stress-related metabolites across different diseases, but no unified metabolic signature has emerged across pain types.

Metabolomic studies related to Tuina remain limited, but animal experiments have documented changes in local muscle metabolic profiles after mechanical stimulation. In a model of delayed-onset muscle soreness, Tuina altered transcriptomic and untargeted metabolomic features of muscle tissue over the course of intervention, involving pathways related to lipid and energy metabolism (Liu et al., 2025b). Another study of massage in rats with skeletal muscle spasm identified clear differences in local skeletal-muscle metabolites together with mitochondrial and energy-metabolism-related changes (Sun et al., 2026). These studies show that manual stimulation can be accompanied by alterations in local metabolic state, but current evidence is concentrated in animal models and stimulated tissues, with little work simultaneously covering blood, feces, and pain phenotypes.

Findings from untargeted metabolomics are also influenced by analytical platform, sample processing, quality control, and the confidence of metabolite annotation. LC–MS provides broad chemical coverage and is widely used in untargeted metabolomic studies, although different chromatographic and ionization conditions differ in their coverage of lipids, bile acids, amino acids, and other polar compounds. Structural identification remains a major limitation of untargeted metabolomics, and high-confidence results typically require integration of tandem mass spectrometry, comparison with authentic standards, and subsequent targeted quantification (Kodra et al., 2022). Recent quality-control consensus also emphasizes the importance of reference samples, pooled QC samples, batch monitoring, and standardized metabolite annotation for reproducibility (Mosley et al., 2024). Untargeted metabolomics is therefore most useful for characterizing potential post-Tuina shifts in the metabolic profile and for providing a basis to identify metabolites with more stable biological significance.

### 5.2 Tuina-related host metabolic changes: energy, lipid, and amino acid metabolism

Metabolic changes after Tuina show clear tissue and temporal characteristics. Longitudinal observations in animal models of delayed-onset muscle soreness indicate that local gene expression and metabolite profiles can change across stages of Tuina intervention (Liu et al., 2025b). In neuropathic pain models, the analgesic and neuroinflammatory responses to Tuina also show a degree of temporal accumulation (Wu Z. et al., 2024). These findings suggest that a metabolic profile measured at a single time point may reflect a combination of immediate mechanical stimulation, tissue recovery, and cumulative treatment effects, and that different sampling times may represent biologically distinct states.

Metabolomic research in chronic pain frequently implicates energy, lipid, and amino acid metabolism. Systematic reviews indicate that patients with chronic pain may show alterations in amino acids, tricarboxylic acid cycle intermediates, lipids, and acylcarnitines, although the direction and magnitude of these changes differ among diseases (Teckchandani et al., 2021; Aroke and Powell-Roach, 2020). In musculoskeletal pain, local energy availability, mitochondrial function, fatty acid oxidation, and lactate metabolism may be linked to muscle stress and nociceptor state. Energy-metabolic, lipid-metabolic, and mitochondrial changes observed in animal studies of Tuina or massage are consistent with this background (Sun et al., 2026; Liu et al., 2025b). At present, however, these findings mainly reflect local tissue metabolic responses, and their relationship to systemic metabolism and gut-microbiota-derived metabolites has not been adequately defined.

Different sample types provide metabolomic information with different biological meanings. Blood metabolites more closely reflect the systemic circulation, fecal metabolites capture metabolic outputs jointly generated by the host and microbiota within the intestinal lumen, and metabolites measured in muscle, spinal cord, or brain are more useful for localizing tissue-specific responses. Previous studies have shown that the fecal metabolome is strongly influenced by gut microbial composition and can serve as a functional layer linking community structure with host phenotypes (Zierer et al., 2018). In Tuina-related research, local tissue metabolic changes, circulating metabolic signals, and fecal co-metabolites currently derive largely from separate studies. Whether changes across these sample compartments occur synchronously and show stable relationships with pain improvement is a key question for understanding the relationship between host metabolic responses and gut microecology.

### 5.3 Gut-derived co-metabolites and pain regulation

Metabolites jointly produced by the gut microbiota and host form an important signaling interface between microecology and neural, immune, and peripheral tissues. Chronic pain research has focused particularly on SCFAs, bile acids, and tryptophan derivatives. These molecules reflect microbial metabolic activity but can also influence pain-related biology through host receptors, immune cells, and neural pathways (Santoni et al., 2021). The functional direction of a metabolite depends on its source, concentration, receptor distribution, and disease context; therefore, an increase or decrease in a single metabolite is insufficient to determine its role in pain.

Among SCFAs, butyrate is supported by a relatively complete microbiota-metabolite-receptor-neural circuit. *Roseburia intestinalis* is reduced in patients with neuropathic pain, and supplementation of this bacterium in animal experiments can attenuate pain hypersensitivity after nerve injury. Further work showed that butyrate produced by *R. intestinalis* activates GPR41 on vagal neurons, enhances afferent signaling to the nucleus tractus solitarius, and subsequently modulates a nucleus tractus solitarius-central amygdala circuit. Deletion of vagal Gpr41 markedly attenuated the analgesic effect (Jiang et al., 2025). This study connects a defined bacterial strain, butyrate, GPR41, and a central pain circuit, providing relatively direct functional evidence that gut-derived metabolic signals can participate in pain regulation.

Microbial bile acid transformation can also influence host pain-related signaling. Animal studies show that activation of TGR5 or FXR in the spinal dorsal horn reduces mechanical hypersensitivity after nerve injury and is accompanied by changes in neuroinflammation (Wu et al., 2023). More recent work found altered bile acid profiles and FXR signaling in neuropathic pain models and showed that hyodeoxycholic acid (HDCA) can improve pain behavior by activating FXR, together with recovery of intestinal barrier function, gut microbiota, and inflammatory status (Lin et al., 2026). These findings suggest that the bile acid environment shaped in part by intestinal microorganisms can interface with neuroimmune regulation through host bile acid receptors, although the direction of effect of individual bile acids remains dependent on receptor distribution and tissue context.

Signaling derived from tryptophan metabolism is more complex. Gut microbiota can directly or indirectly regulate three major branches involving serotonin, kynurenine, and indole derivatives (Agus et al., 2018). Microbiota-derived indoles can act as ligands of the aryl hydrocarbon receptor (AhR) and participate in mucosal immunity and host-microbial communication (Hubbard et al., 2015). In human studies, tryptophan-related metabolites have also been associated with the severity of osteoarthritis pain, although the direction and strength of these associations vary among studies (Meléndez-Oliva et al., 2025). Compared with butyrate and some bile acid pathways, tryptophan metabolites currently show broader, multibranch associations with chronic pain, and their specific receptor and functional chains remain dependent on the pain condition and metabolic branch involved.

These microbiota-metabolite-receptor relationships are derived mainly from microbiome and pain-mechanism research, and there is currently no evidence that the same pathways directly mediate Tuina analgesia. Existing Tuina studies have also not simultaneously tracked microbial functional genes, fecal and circulating metabolites, corresponding host receptors, and pain phenotypes. Accordingly, the biological significance of post-Tuina changes in SCFAs, bile acids, or tryptophan metabolites needs to be interpreted in relation to their microbial functional sources, measured metabolite levels, and corresponding host pathways.

### 5.4 Gut-derived metabolic signals and central pain regulation

Gut-derived metabolic signals can influence the central nervous system through multiple routes. Some small molecules enter the circulation and directly or indirectly affect blood–brain barrier integrity and neural cell function, whereas other metabolites act mainly through peripheral immune cells, enteroendocrine signals, and vagal afferents to modify central neural activity (Parker et al., 2020; Jameson et al., 2025). Selected microbial metabolites within the intestinal lumen can regulate vagal activity through receptor-dependent signaling, indicating that metabolic changes can exert distal central effects through neural afferents even without directly entering brain tissue (Jameson et al., 2025). This provides a potential functional connection between changes in gut microecology and central pain processing.

In chronic pain, circulating metabolic signals, peripheral immune responses, and vagal afferent input may all influence microglia, neuroinflammation, and central sensitization. Metabolites such as tryptophan derivatives can also act at multiple levels through immune and neurotransmitter systems (Kondo et al., 2024). However, existing Tuina studies have not continuously measured fecal or circulating metabolites together with central neuroimmune indices and pain phenotypes, and direct validation of gut-derived metabolic signaling in central analgesia after Tuina is lacking. Microbiome and pain studies provide the relevant biological background, but different metabolites do not reach or influence the central nervous system through identical routes, and their effects are also shaped by blood–brain barrier status, peripheral immunity, and receptor distribution.

### 5.5 Bidirectional gut-muscle metabolic and inflammatory signaling in the skeletal muscle pain microenvironment

Changes in gut microecology can influence skeletal muscle through circulating metabolic and immune signals. SCFAs, bile acids, and other microbiota-related metabolites are linked to systemic inflammation, glucose and lipid metabolism, mitochondrial function, and oxidative stress, all of which can alter muscle energy supply and the local immune microenvironment (Xu and He, 2025; Li et al., 2024). Chronic musculoskeletal pain itself is also frequently accompanied by changes in local microcirculation, mitochondrial quality control, oxidative stress, and inflammation (Wu K. et al., 2024). After entering the circulation, gut-derived signals may further affect this local environment through metabolic, immune, and endothelial processes, creating a potential link between gut microecology and muscle pain states.

Skeletal muscle also acts as an active endocrine and metabolic signaling organ. Muscle can release myokines such as IL-6 and irisin/FNDC5 and generate lactate, lipid mediators, and other metabolic signals that enter the circulation and participate in systemic inflammation, energy metabolism, and distal organ regulation (Saponaro et al., 2024). Muscle-derived signals and the gut have been increasingly linked in research on exercise, aging, and metabolism, where changes in selected myokines and metabolic states occur alongside changes in intestinal barrier function, mucosal immunity, and microbial composition (Xu and He, 2025; Li et al., 2024). Transient IL-6 release associated with muscle contraction occurs in a different physiological context from sustained IL-6 elevation in chronic inflammation, while myokines such as irisin also show potential to modulate inflammation and the intestinal environment. Skeletal muscle may therefore both receive gut-derived metabolic and inflammatory signals and influence intestinal and systemic homeostasis through myokines and metabolites.

Animal studies of Tuina and massage have documented changes in local skeletal-muscle energy metabolism, mitochondrial function, and metabolite profiles (Sun et al., 2026; Liu et al., 2025b). These changes may arise directly from mechanical stimulation of local tissues and may also coexist with circulating and neuroendocrine responses. No study has yet continuously demonstrated the complete sequence of Tuina mechanical stimulation, altered muscle-derived signals, changes in gut microecology, metabolite changes, and pain improvement. Whether gut-derived metabolites, muscle-derived signals, and local muscle metabolism form bidirectional feedback, and how much such feedback contributes to Tuina analgesia, remain unresolved components of the brain-gut-muscle axis.

### 5.6 Identification and functional confirmation of differential metabolites

The large number of differential features generated by untargeted metabolomics initially reflects differences in metabolic profiles between samples, and biological interpretation depends on the reliability of metabolite structural identification. Retention time, accurate mass, and tandem mass spectral matching can improve annotation confidence, but candidate structures obtained from database matching may still be affected by isomers and annotation errors. Comparison with authentic standards and subsequent targeted quantification can further confirm the identity and actual concentration of candidate metabolites, while quality-control samples, batch monitoring, and standardized data processing help reduce platform-related technical variation (Kodra et al., 2022; Mosley et al., 2024). In Tuina research, the number of differential metabolites or pathway-enrichment results alone cannot establish a functional relationship between a specific metabolic pathway and analgesia (Table 2).

**Table 2:** Representative studies supporting microbiome–metabolite–host interactions relevant to Tuina analgesia.

Study context/model/Reference	Intervention/exposure	Microbial feature/function	Specific mediator	Receptor/pathway	Main biological/pain effect	Relationship to Tuina analgesia
Ulcerative colitis mouse model (Wang et al., 2022)	Tuina	Restored microbial diversity; increased Firmicutes/Lachnospiraceae-related taxa; host colonic proteomic changes	No specific microbiota-derived metabolite validated	Biotin metabolism, Notch signaling, linoleic-acid metabolism and autophagy identified by proteomics	Improved body weight, disease activity, colon histology and intestinal inflammation alongside microbiota/proteomic changes	Direct Tuina study, but a non-pain disease model; no specific microbiota-derived mediator was functionally validated.
Insomnia rat model (Liu et al., 2025a)	Abdominal massage	16S-defined microbial shifts; PICRUSt2 implicated carbohydrate/amino-acid metabolism and transcriptional regulation	Brain–gut peptides; no specific microbial metabolite established	Predicted microbial functions/brain–gut peptide regulation	Improved sleep-related outcomes with changes in inflammatory factors, brain–gut peptides and microbial community structure	Manual intervention in a non-pain model; supports a possible gut response to mechanical stimulation.
Knee osteoarthritis patients (Guo et al., 2025)	Electroacupuncture + Tuina	Altered gut microbiota composition	Not established	No specific microbiota–metabolite–receptor pathway validated	Microbiota changes accompanied improvements in WOMAC-related pain/function outcomes	Pain-related human study, but a combined intervention; the independent contribution of Tuina cannot be isolated.
Neuropathic pain (Jiang et al., 2025)	*Roseburia intestinalis* supplementation/mechanistic experiments	*R. intestinalis*; butyrate-producing function	Butyrate	GPR41; vagal afferent/NTS–CeA circuit	Reduced neuropathic pain hypersensitivity; receptor/pathway intervention supported functional involvement	Strong pain-mechanism study; not direct Tuina evidence. Provides a specific microbiota–metabolite–receptor example.
Neuropathic pain (Wu et al., 2023)	Pharmacological activation of bile-acid receptors	Bile-acid signaling; microbial origin not directly tested in this experiment	Bile-acid receptor agonism	TGR5 and FXR in the spinal dorsal horn	Reduced mechanical hypersensitivity and neuroinflammatory responses	Pain-mechanism study supporting bile-acid receptor involvement; indirect to Tuina and gut-microbiota regulation.
Neuropathic pain (Lin et al., 2026)	HDCA intervention	Gut microbiota/bile-acid profile changes	Hyodeoxycholic acid (HDCA)	FXR signaling	Improved neuropathic pain behavior with changes in intestinal barrier and inflammatory status	Pain-mechanism study linking a bile acid to host receptor signaling; not direct Tuina evidence.
Fibromyalgia (Cai et al., 2025)	Patient-derived microbiota transplantation to germ-free mice	Fibromyalgia-associated microbial community	No single mediator established	Immune and metabolic alterations after microbiota transfer	Transferred pain hypersensitivity with immune/metabolic changes	Functional microbiome–pain evidence; not a Tuina study. Supports a causal role of microbiota in pain phenotype formation.
Skeletal-muscle spasm rat model (Sun et al., 2026)	Massage	Local skeletal-muscle metabolomic changes	136 differential metabolites; inositol/lipid-metabolism-related signals	Mitochondrial and energy-metabolism restoration	Reduced muscle spasm pain and improved metabolic/mitochondrial features	Direct manual-stimulation evidence for muscle metabolic responses; no gut-microbiota involvement was demonstrated.
Delayed-onset muscle soreness/EIMD rat model (Liu et al., 2025b)	Tuina	Local muscle transcriptomic and untargeted-metabolomic changes	Multiple differential metabolites; no single mediator validated	Inflammatory mediator regulation of TRP channels, cAMP signaling, ferroptosis and muscle-repair pathways	Increased mechanical withdrawal threshold, reduced CK-MM and improved muscle repair with time-dependent molecular/metabolic changes	Direct Tuina metabolic evidence in a pain-related muscle model; gut microbial mediation was not tested.
Ulcerative colitis mouse model (Huangfu et al., 2021)	Exogenous irisin	Altered intestinal microbiota accompanied anti-inflammatory effects	Irisin	Inflammatory/intestinal-microenvironment regulation	Attenuated colonic inflammation with microbiota changes	Physiological support for a muscle-derived signal affecting the gut; neither Tuina nor pain was studied.

Synchronous changes in metabolites and pain phenotypes remain associative unless supported by more direct biological validation. Microbiome and pain studies have altered pain phenotypes through metabolite supplementation, receptor blockade, or genetic intervention. For example, functional experiments on the butyrate-GPR41 pathway show that blocking a key receptor can markedly attenuate the corresponding analgesic effect (Jiang et al., 2025). For metabolites of microbial origin, microbiota transplantation, microbiota depletion, or intervention with a specific strain can further determine whether the metabolic change depends on the microbial community. Clinical studies rely more heavily on longitudinal repeated measurements, targeted quantification, and replication across cohorts to assess the stability of candidate metabolite associations with therapeutic response (Metwaly et al., 2025).

Current metabolomic studies related to Tuina mainly show changes in metabolic profiles and enriched pathways after intervention, whereas functional studies simultaneously connecting microbial function, specific metabolites, host receptors, and pain phenotypes remain scarce. Reproducible detection of candidate metabolites across samples, concordant changes with corresponding microbial functions, and altered pain phenotypes after functional intervention can progressively strengthen mechanistic interpretation. Association nodes identified by integrating metagenomics and metabolomics can thereby be narrowed to molecules with clearer metabolic origins and host targets, providing a basis for subsequent integration of multi-omics mechanistic networks.

## 6 Multi-omics integration of microecological-metabolic links related to Tuina analgesia

### 6.1 Integrated analysis of metagenomics and untargeted metabolomics

Metagenomics and untargeted metabolomics provide complementary information on microecology. Metagenomics characterizes microbial composition, strain-level features, functional genes, and metabolic pathway potential, whereas metabolomics reflects the actual metabolic state jointly shaped by the host and microbiota. When integrated, the two datasets can be used to determine whether changes in microbial functions occur in parallel with corresponding metabolites and to analyze their relationships with inflammation, neuroimmune status, and pain phenotypes (Yang S. Y. et al., 2025; Puig-Castellví et al., 2023). For example, correspondence between SCFA-production genes and measured SCFA levels, between bile-acid-transforming functions and bile acid profiles, or between tryptophan-metabolism genes and indole metabolites can add a functional layer of interpretation beyond microbial abundance alone.

Connections across multi-omics datasets can be identified through microbe-metabolite correlation analysis, matching of functional genes with metabolic pathways, co-occurrence networks, and multivariable models (Puig-Castellví et al., 2023; Ning et al., 2023). In Tuina-related research, concordant changes among microbial taxa, functional genes, metabolites, pain intensity, pressure pain threshold, inflammatory indices, or other clinical phenotypes may define cross-level associations worthy of further validation. However, such associations remain vulnerable to limited sample size, confounding, batch effects, and collinearity among high-dimensional variables, and the strength of a network connection does not directly represent biological dependence.

Different study designs provide different levels of support for mechanistic interpretation. A microbiota-metabolite association observed at a single time point primarily reflects synchronous variation; confidence increases if the same relationship shows a stable temporal pattern in longitudinal sampling and is reproduced by targeted metabolite quantification or in independent samples. Mediation analysis can assess whether a candidate metabolite lies within a statistical relationship between the intervention and a pain phenotype, but it cannot replace functional experiments. Microbiota transplantation, supplementation with specific strains, metabolite intervention, and receptor blockade can further test whether key nodes have functional roles (Metwaly et al., 2025). For example, the association between *Roseburia intestinalis*-derived butyrate and neuropathic pain has been supported across strain supplementation, metabolite measurement, and GPR41-related intervention (Jiang et al., 2025). In Tuina analgesia, however, current research remains dominated by omics associations, with little continuous validation extending from microbial function and metabolite changes to host receptors and pain phenotypes.

### 6.2 Potential signaling links between Tuina mechanical stimulation and the intestinal microenvironment

After acting on the skin, fascia, and skeletal muscle, Tuina generates mechanosensory and proprioceptive input together with changes in local tissue and neurophysiological states. Manual therapy studies have reported alterations in sympathetic- or parasympathetic-related indices after some interventions, but findings differ across techniques, measured outcomes, and study quality, and neither the direction nor the clinical meaning of autonomic effects has been consistently established (Keter et al., 2025; Roura et al., 2021). Autonomic activity is closely linked to intestinal motility, secretion, mucosal barrier function, and local immune status, providing a possible neural connection between peripheral mechanical stimulation and the intestinal environment. Animal experiments further show that stress-related neural circuits can alter microbial niches through duodenal glands and mucus secretion, demonstrating that host neural activity itself can reshape the intestinal microenvironment and microbial composition (Chang et al., 2024). Continuous studies directly showing that Tuina changes gut microbiota through autonomic regulation and thereby produces analgesia are currently lacking.

Distal biological changes after mechanical stimulation may also involve circulating signals beyond the autonomic nervous system. Skeletal muscle has endocrine and metabolic functions and can release myokines such as IL-6 and irisin, as well as lactate, lipid mediators, and other metabolites involved in interorgan communication (Saponaro et al., 2024; Sabaratnam et al., 2022). Skeletal-muscle-derived extracellular vesicles can also carry proteins, lipids, miRNAs, and other bioactive cargo into the circulation, and distal tissue effects have been observed in exercise and metabolic research (Ni et al., 2023). Animal studies of Tuina and massage have identified changes in local skeletal-muscle energy metabolism and metabolite profiles (Sun et al., 2026; Liu et al., 2025b), but it remains unknown whether these local responses further alter circulating myokines, extracellular vesicles, or miRNAs, or whether such signals affect the intestinal microenvironment.

Systemic responses to Tuina may therefore involve autonomic, neuroimmune, and circulating metabolic signals in parallel, while changes in gut microecology may be influenced by multiple host states. Extracellular vesicles and their miRNA cargo, myokines, and muscle-derived metabolites are currently proposed mainly on the basis of exercise physiology and cross-organ communication research, and their roles in Tuina analgesia remain exploratory. No study has yet continuously measured mechanical stimulation parameters, autonomic activity, circulating muscle-derived signals, intestinal barrier and microbial functions, metabolites, and pain phenotypes within the same experimental framework.

### 6.3 Candidate cascades linking microbial function, metabolic signals, and host responses

Multi-omics data allow microbial functions, metabolite changes, and host responses to be considered within the same biological chain, with SCFA-related pathways currently supported by relatively clear experimental evidence. SCFA-producing microbial communities and their functional genes can influence the formation of metabolites such as butyrate and propionate, which can in turn participate in intestinal barrier regulation, immune homeostasis, and neural signaling through receptors including GPR41 and GPR43. *Roseburia intestinalis*, for example, produces butyrate that can influence neuropathic pain through GPR41-related vagal signaling, providing experimental support for a continuous link among microbial function, metabolites, host receptors, and pain phenotypes (Jiang et al., 2025). If Tuina is followed by concordant changes in corresponding microbial functions, SCFA levels, inflammatory indices, and pain phenotypes, this would suggest possible involvement of the pathway in systemic responses to the intervention, but omics associations alone would not establish mediation.

Bile acid and tryptophan metabolism provide two additional biologically plausible cross-level links. Gut microorganisms participate in bile acid deconjugation and secondary bile acid formation, and the resulting metabolites can influence immunity, energy metabolism, and neural function through receptors such as FXR and TGR5. Roles for these receptors in neuropathic pain are supported by animal experiments (Lin et al., 2026; Wu et al., 2023). Tryptophan can enter several branches involving serotonin, kynurenine, and microbiota-derived indoles; indole-AhR signaling contributes to intestinal barrier and immune regulation and may also be linked to neuroinflammation and pain states (Meléndez-Oliva et al., 2025; Kondo et al., 2024). Compared with SCFA pathways, tryptophan-related mechanisms in chronic pain are more dependent on the specific metabolic branch and disease context, leaving their functional relevance to Tuina particularly open.

Changes in lipid inflammatory mediators and energy metabolism more often reflect integrated host-tissue responses to mechanical stimulation, inflammation, and pain. Arachidonic acid derivatives, oxidized lipids, and phospholipid metabolites can influence inflammation and nociceptor excitability, while local impairment of energy metabolism, oxidative stress, and mitochondrial dysfunction are also associated with persistent myogenic pain (Wu K. et al., 2024; Sun et al., 2026). These metabolic changes may coexist with gut microecological alterations but may also arise directly from skeletal-muscle mechanical responses, autonomic and neuroendocrine regulation, or changes in activity, sleep, emotion, diet, and concomitant therapy. Multi-omics changes after Tuina therefore do not imply that all metabolic signals are initiated by the gut microbiota. Microbial functions, host metabolism, and brain or muscle target-organ responses are more likely to form several interconnected pathways, whose direction and relative contribution need to be evaluated through temporal analysis, targeted quantification, and functional intervention.

### 6.4 Multi-omics candidate biomarkers related to Tuina response

The clinical manifestations of chronic pain and responses to treatment are influenced by pain sensitization, inflammatory status, sleep and emotional state, metabolic background, gut microecology, and other factors, making it difficult for a single biological measure to capture this heterogeneity (Pak et al., 2024; Curatolo et al., 2025). Metagenomic and metabolomic data can provide candidate features at multiple levels, including microbial functions related to SCFA production, bile acid transformation, and tryptophan metabolism; SCFAs, bile acid derivatives, indoles, and lipid metabolites; and clinical phenotypes such as pain intensity, pressure pain threshold, functional status, and inflammatory markers. Stable relationships among features from different levels within the same individuals may be more informative for describing variation in biological responses to Tuina than any single taxon or metabolite.

Systematic studies comparing multi-omics differences between responders and nonresponders to Tuina are still lacking. In chronic low back pain and fibromyalgia, clinical phenotypes are beginning to be integrated with multi-omics measures to identify feature combinations associated with pain heterogeneity and treatment response (Curatolo et al., 2025; Sluka et al., 2023). Similar approaches could examine whether microbial functions, metabolic profiles, and clinical responses form reproducible patterns before and after Tuina. At present, however, no prospectively validated microbiome or metabolite biomarker panel can predict response to Tuina. These measures therefore mainly represent candidate response-related features, and their predictive performance and range of applicability require confirmation in independent populations.

The high dimensionality of multi-omics data also creates opportunities for machine-learning analysis, but model performance is readily affected by sample size, batch effects, feature redundancy, and overfitting. Cross-cohort microbiome classification studies show that performance can decline substantially when the same model is applied to different cohorts, highlighting the importance of external validation for assessing stability (Li et al., 2023). Tuina adds intervention-specific sources of variation, including manipulation type, stimulation dose, practitioner, and pain condition, which may further influence relationships between multi-omics features and therapeutic response. At present, the value of multi-omics candidate biomarkers lies mainly in describing treatment-response heterogeneity and prioritizing biological features for further validation, while their role in clinical prediction remains exploratory.

### 6.5 Causal validation of multi-omics associations

Correlations among microbiota, functional genes, metabolites, and pain phenotypes in multi-omics networks primarily indicate synchronous variation across biological levels and do not by themselves demonstrate functional dependence or mediation (Metwaly et al., 2025). The interpretation of such relationships can be progressively strengthened through study design. Cross-sectional covariation identifies candidate nodes; longitudinal observation can clarify temporal relationships among microbial functions, metabolites, and pain changes; targeted metabolite quantification and replication in independent samples can assess reproducibility; and when a candidate metabolite shows stable associations with the intervention, microbial function, and pain phenotype, mediation and pathway-consistency analyses can be used to estimate its possible position in the network. These approaches strengthen mechanistic inference, but statistical temporal order and mediation cannot replace direct functional validation.

Functional perturbation in animal and cellular models can further determine whether key nodes participate directly in pain regulation. Microbiota depletion, fecal microbiota transplantation, germ-free models, and supplementation with specific strains can test whether changes in microbial communities or candidate organisms alter pain phenotypes. At the metabolic level, metabolite supplementation, metabolic-pathway intervention, and receptor blockade can further test the functional effects of candidate metabolic signals (Metwaly et al., 2025). Transplantation of microbiota from patients with fibromyalgia into germ-free mice can induce pain hypersensitivity and metabolic changes, providing functional support for microbial participation in pain phenotypes (Cai et al., 2025). Studies of *Roseburia intestinalis* and its butyrate product further establish links among a strain, metabolite, host receptor, and pain changes through GPR41-related intervention (Jiang et al., 2025). These experiments show that stable omics associations approach mechanistic biological relationships only when they are corroborated by functional perturbation.

Causal interpretation in Tuina research is also constrained by the reproducibility of the intervention itself. Manipulation type, treatment site, stimulation intensity, frequency, treatment duration, and practitioner-related differences can all change the actual mechanical exposure and thereby influence neural, immune, microecological, and metabolic responses (Zhang et al., 2023). Thus, even when stable associations emerge among microbiota, metabolites, and pain improvement, Tuina stimulation parameters and their temporal changes must be considered when judging the relationship between these biological changes and the intervention. The field currently lacks studies that continuously cover mechanical stimulation, autonomic or circulating signals, microbial functions, metabolites, host receptors, and pain phenotypes and then subject these links to functional perturbation. Existing multi-omics findings are mainly useful for identifying nodes worthy of further testing, while a complete mechanism will require mutually supportive evidence from longitudinal observation, targeted replication, and functional experiments (Figure 3).

**Figure 3:**
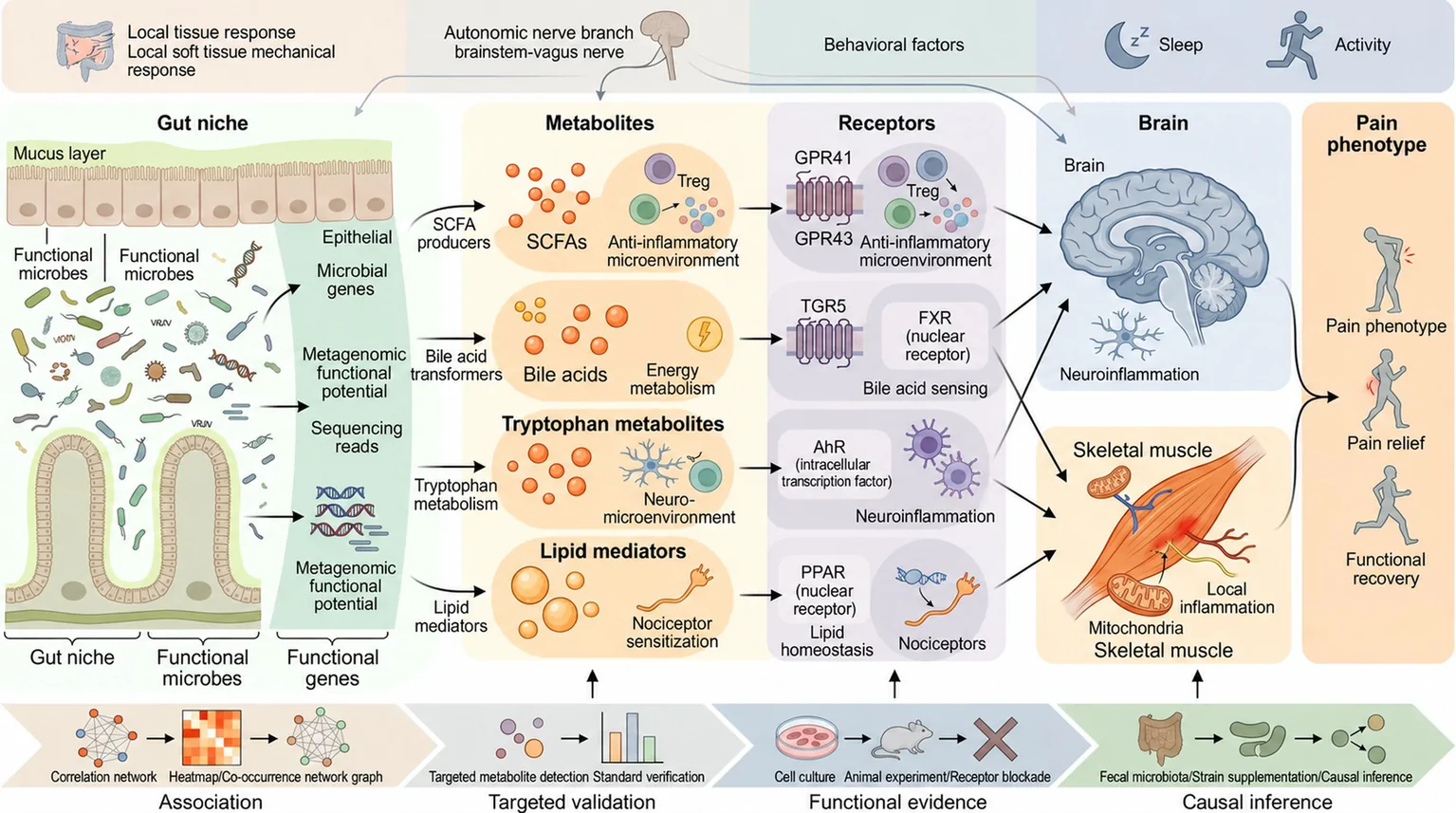
Multi-omics candidate network of microbial functions, metabolic signals, and host responses related to Tuina analgesia. The network integrates gut microbial functions, short-chain fatty acids, bile acids, tryptophan-derived metabolites, and lipid-related signals with host pathways including GPR41/GPR43, FXR, TGR5, AhR, and PPAR, together with responses in the brain and skeletal muscle. The lower panel summarizes progression from omics associations to targeted validation, functional experiments, and causal testing. Several candidate links still require direct functional validation in Tuina studies. SCFA/SCFAs, short-chain fatty acid(s); GPR41/43, G protein-coupled receptor 41/43; FXR, farnesoid X receptor; TGR5, Takeda G protein-coupled receptor 5; AhR, aryl hydrocarbon receptor; PPAR, peroxisome proliferator-activated receptor; Treg, regulatory T cell.

## 7 Standardization, stratification, and clinical translation of multi-omics research on Tuina analgesia

### 7.1 Standardization of Tuina intervention and multi-omics workflows

The actual intervention exposure produced by Tuina is influenced by technique type, treatment site, force, rhythm, duration, treatment frequency, depth of tissue engagement, and practitioner-related factors, so mechanical stimulation can differ substantially even under the same label of “Tuina.” STRICTOTM incorporates technical details of Tuina/massage, treatment protocols, practitioner background, comparator interventions, and safety-related information into a standardized reporting framework, providing a systematic basis for comparing intervention exposure across studies (Zhang et al., 2023). In multi-omics research, parameters such as pressure or applied force, rhythm, treatment duration, and frequency may vary in parallel with neuroimmune, metabolic, and microecological responses; the degree to which intervention parameters are quantified therefore directly affects the interpretability of omics findings across individuals and studies.

Sample collection and host background represent another major source of variation. Gut transit time, bowel frequency, and stool consistency can substantially affect microbial composition and the metabolic environment, while dietary pattern and recent food intake also modify microbial functions and metabolic outputs (Procházková et al., 2023). Patients with chronic pain are commonly exposed to NSAIDs, opioids, antidepressants, or other medications, while antibiotics and probiotics can cause more direct perturbations of the microbiota. Age, sex, BMI, sleep, physical activity, and comorbidities may likewise be associated with both pain and microecological features. Sampling time can add circadian variation, and studies have shown that samples collected at different times of day can yield different microbiome comparisons within the same experimental setting (Allaband et al., 2024). Collection time, preservation and freezing conditions, DNA extraction, sequencing depth, and experimental batch can introduce additional variation in fecal, blood, and local tissue samples. In metabolomics, sample processing, internal standards, pooled quality-control samples, and batch monitoring similarly affect measurement stability (Mosley et al., 2024).

Statistical analysis of omics data needs to account for these clinical and technical variables. MaAsLin2 can incorporate multiple covariates when modeling associations of microbial taxa, functional genes, or metabolic features with clinical phenotypes and supports repeated-measures and longitudinal data structures (Mallick et al., 2021). For compositional microbiome data, ANCOM-BC can correct sampling-fraction bias, while ANCOM-BC2 further supports multigroup comparisons, covariate adjustment, and repeated-measures analysis (Lin and Peddada, 2020; Lin and Peddada, 2024). Repeated measurements from the same participant can also be analyzed with mixed-effects models to account for within-subject correlation, and multiple testing correction is required for high-dimensional features. Batch differences, outlier handling, and alternative covariate sets may affect final associations, so sensitivity analyses can be used to examine the robustness of key findings under different analytical specifications.

Complete reporting of the study process and analytical details also determines whether findings can be reproduced and compared. STORMS covers study design, participant characteristics, sample processing, bioinformatics workflows, and statistical analysis in microbiome research and emphasizes the recording of diet, medication, and other host variables (Mirzayi et al., 2021). Integrating STRICTOTM reporting of the Tuina intervention with microbiome and metabolome sampling and analytical standards can document mechanical stimulation exposure, host confounders, and omics-related technical variation within the same study. Together, these elements determine whether Tuina-related microecological and metabolic changes can be interpreted consistently across time points, practitioners, and study populations (Table 3). Table 3 also summarizes major microbiome confounders (Procházková et al., 2023; Tito et al., 2024; Perler et al., 2023), sample collection and storage (Mirzayi et al., 2021; Isali et al., 2024), metagenomics (Yang S. Y. et al., 2025; Mirzayi et al., 2021; Bai et al., 2025), and multi-omics integration (Puig-Castellví et al., 2023; Muller et al., 2024).

**Table 3:** Key intervention, sampling, analytical, and reporting considerations for multi-omics studies of Tuina.

Domain/Reference	Key variables	Main analytical/reporting considerations	Relevant guideline/method
Tuina intervention (Tune et al., 2023; Zhang et al., 2023)	Technique, treatment site, force/pressure, rhythm, duration, frequency, tissue depth, practitioner, concomitant therapy	Describe the intervention in sufficient detail; quantify mechanical exposure when feasible; document practitioner consistency and control procedures.	STRICTOTM; quantitative manipulation recording
Clinical phenotyping (Mak et al., 2024; Zhou et al., 2023)	Pain type and duration, pain intensity, pressure pain threshold, disability/function, sleep/emotion, medications, concomitant rehabilitation	Use consistent outcome definitions and time points; record clinical factors that may modify treatment response or omics profiles.	Standardized clinical outcome reporting
Microbiome confounders (Procházková et al., 2023; Tito et al., 2024; Perler et al., 2023)	Diet, recent dietary intake, gut transit time, stool consistency/frequency, antibiotics, probiotics, NSAIDs/opioids, sleep, physical activity, age, sex, BMI, comorbidities	Collect key covariates prospectively and include them in matching, multivariable adjustment and sensitivity analyses as appropriate.	Confounder-aware microbiome analysis
Sample collection and storage (Mirzayi et al., 2021; Isali et al., 2024)	Feces, blood and/or local tissue; collection timing, relation to treatment, preservation, freezing, storage duration, contamination control	Keep collection and storage procedures consistent across visits and centers; retain complete sample metadata.	STORMS; microbiome sample-collection guidance
Metagenomics (Yang S. Y. et al., 2025; Mirzayi et al., 2021; Bai et al., 2025)	DNA extraction, sequencing platform/depth, host-sequence removal, taxonomic annotation, strain-level analysis, functional annotation databases	Report preprocessing and annotation workflows; distinguish taxonomic composition from functional-gene/pathway potential; monitor technical batch effects.	KEGG/MetaCyc/eggNOG/HUMAnN; standardized metagenomic workflows
Untargeted metabolomics (Kodra et al., 2022; Mosley et al., 2024)	Analytical platform, internal standards, pooled QC, batch drift, feature filtering, MS/MS matching, annotation confidence, authentic standards, targeted confirmation	Use QC samples and batch monitoring; report identification confidence; confirm key metabolites with authentic standards and targeted quantification when possible.	LC–MS metabolomics QA/QC and metabolite-identification practice
Statistical analysis (Mallick et al., 2021; Lin and Peddada, 2020, 2024)	Covariate-adjusted feature associations, compositional differential abundance, repeated measures, multiple testing, batch effects, sensitivity analyses	Use multivariable models for clinical covariates; compositional methods for differential abundance; mixed/repeated-measures models for longitudinal data; control FDR and test robustness.	MaAsLin2; ANCOM-BC/ANCOM-BC2
Multi-omics integration (Puig-Castellví et al., 2023; Muller et al., 2024)	Microbial taxa/strains, functional genes, metabolite profiles, neuroimmune variables and pain phenotypes	Match functional genes with metabolic pathways and measured metabolites; use covariate-adjusted network/module analyses; prioritize reproducible links for targeted or functional validation.	Metagenome–metabolome integration frameworks

### 7.2 Multi-omics candidate features and therapeutic response heterogeneity

Treatment responses in chronic pain show substantial interindividual variability and are related to pain sensitization, inflammatory status, psychosocial factors, sleep, metabolic background, and other biological features (Hutchinson et al., 2025; Fillingim et al., 2025). Gut microecology and metabolic state may form part of this background. Metagenomic and metabolomic studies can capture microbial functions, metabolic signals such as SCFAs and bile acids, and clinical pain and functional phenotypes within the same analysis, providing multidimensional information for comparing biological differences among patients with different treatment responses. Studies in chronic low back pain and fibromyalgia have begun to integrate clinical characteristics with multi-omics measures, but this work remains largely at the stage of feature identification and exploratory model development (Curatolo et al., 2025).

Tuina-related research currently lacks prospectively validated combinations of microbial or metabolic biomarkers. If reproducible differences in microbial functions and metabolic profiles are observed before or during treatment among groups with different responses, these features could help describe therapeutic-response heterogeneity and support subsequent analysis of their relationships with pain sensitization, inflammation, and responses to mechanical stimulation. A single microbial taxon, metabolite, or clinical measure is highly susceptible to individual background and measurement variability. Combining features across several dimensions may provide more information, but stability still needs to be tested across populations and Tuina protocols.

Machine learning can handle high-dimensional, nonlinear multi-omics data and can be used to screen combinations of features related to clinical phenotypes (Rozera et al., 2025). Such models are sensitive to sample size, batch effects, feature selection, and data leakage. Cross-cohort microbiome studies show that high within-cohort performance may decline markedly in external populations, underscoring the need for cross-cohort validation when evaluating model generalizability (Li et al., 2023). Tuina studies also need to account for intervention-specific factors such as manipulation dose, practitioner, pain type, and concomitant therapy. At present, machine learning is better suited to exploring response-related patterns and narrowing candidate features than to clinical response prediction or treatment decision-making.

### 7.3 Lifestyle and microecological supportive strategies

Comprehensive management of chronic pain commonly includes exercise rehabilitation, sleep management, psychological or behavioral approaches, and other lifestyle factors, all of which can also influence gut microecology and host metabolism (Shi and Wu, 2023). Diet is a particularly direct modifier of the microbial environment: dietary fiber, fat composition, and overall dietary patterns can alter microbial structure and metabolic function. For example, the Mediterranean diet has been associated with gut microbial composition and SCFA-related metabolic features (Barber et al., 2023). Exercise and sleep can likewise affect both pain and the intestinal environment through metabolic, immune, and neuroendocrine pathways. During Tuina treatment, these lifestyle factors may therefore serve as components of supportive management while also influencing interpretation of microbiome and metabolome findings.

Prebiotics, probiotics, and interventions targeting specific microbial metabolites represent potential directions in microecological research on chronic pain, but clinical evidence remains limited across pain conditions (Lou et al., 2025). Baseline microbiota, diet, medication use, and disease phenotype may all modify responses to these interventions, and evidence is currently insufficient to support their routine use as adjuncts to Tuina. More direct microbiota-modifying interventions such as fecal microbiota transplantation have stronger biological effects and more complex safety and causal implications; they are currently relevant mainly to tightly controlled mechanistic research or diseases with established clinical indications and should not be inferred as treatment strategies solely from pain-related omics associations (Metwaly et al., 2025).

Lifestyle management and microecological intervention therefore occupy different positions in Tuina analgesia. Diet, exercise, and sleep management already have broader roles in comprehensive chronic pain care, whereas interventions targeting specific taxa or metabolites remain strongly exploratory. Multi-omics findings can help identify microecological or metabolic changes that occur in parallel with pain improvement, but whether those changes justify a dedicated microecological intervention depends on functional validation of the relevant pathways and demonstrable clinical benefit.

### 7.4 Indications and feasibility in clinical translation

The clinical evidence base for Tuina and related manual therapies differs across pain conditions. Systematic evidence on massage for pain shows that most conclusions remain supported by low- or very-low-certainty evidence and that the strength of evidence varies by indication (Mak et al., 2024). For example, manual therapy for knee osteoarthritis has some evidence for improvement in pain and function (Zhu et al., 2024), whereas the overall certainty of evidence for massage in neck pain remains low and clinical benefit is limited in some comparisons (Gross et al., 2024). The clinical meaning of multi-omics findings therefore needs to be interpreted in relation to the specific disease, manual therapy protocol, treatment course, and existing efficacy evidence rather than extrapolated from one pain condition to another.

Clinical feasibility is also influenced by intervention standardization, safety, therapist training, patient adherence, and long-term follow-up. Because Tuina is practitioner-dependent, the same treatment label may correspond to different mechanical exposures, making the technical details, treatment protocol, and practitioner information emphasized by STRICTOTM important for cross-study comparison (Zhang et al., 2023). Multi-omics measurements also involve sample collection, experimental costs, batch variation, and complex data analysis, all of which affect feasibility in large-scale clinical studies. At present, microbiome and metabolome measures are better viewed as additional information for mechanistic studies and analyses of therapeutic-response heterogeneity; their value as routine clinical tests has not been established.

In fields such as chronic low back pain, trial designs have begun to integrate biomarkers, clinical phenotypes, and treatment response within the same framework. The BEST trial, for example, uses extensive phenotyping and biomarker data to explore differences in how patients respond to commonly used treatments, providing a reference for studying patient heterogeneity in pain research (Mauck et al., 2025). Similar designs could be adapted for Tuina by examining mechanical stimulation parameters, microecological functions, and metabolic features alongside conventional efficacy and safety outcomes, although these measures currently have mainly exploratory roles. Only if such features show stable reproducibility in prospective studies and external populations and provide information beyond conventional clinical measures can their stratification or decision-making value be more clearly established (Figure 4).

**Figure 4:**
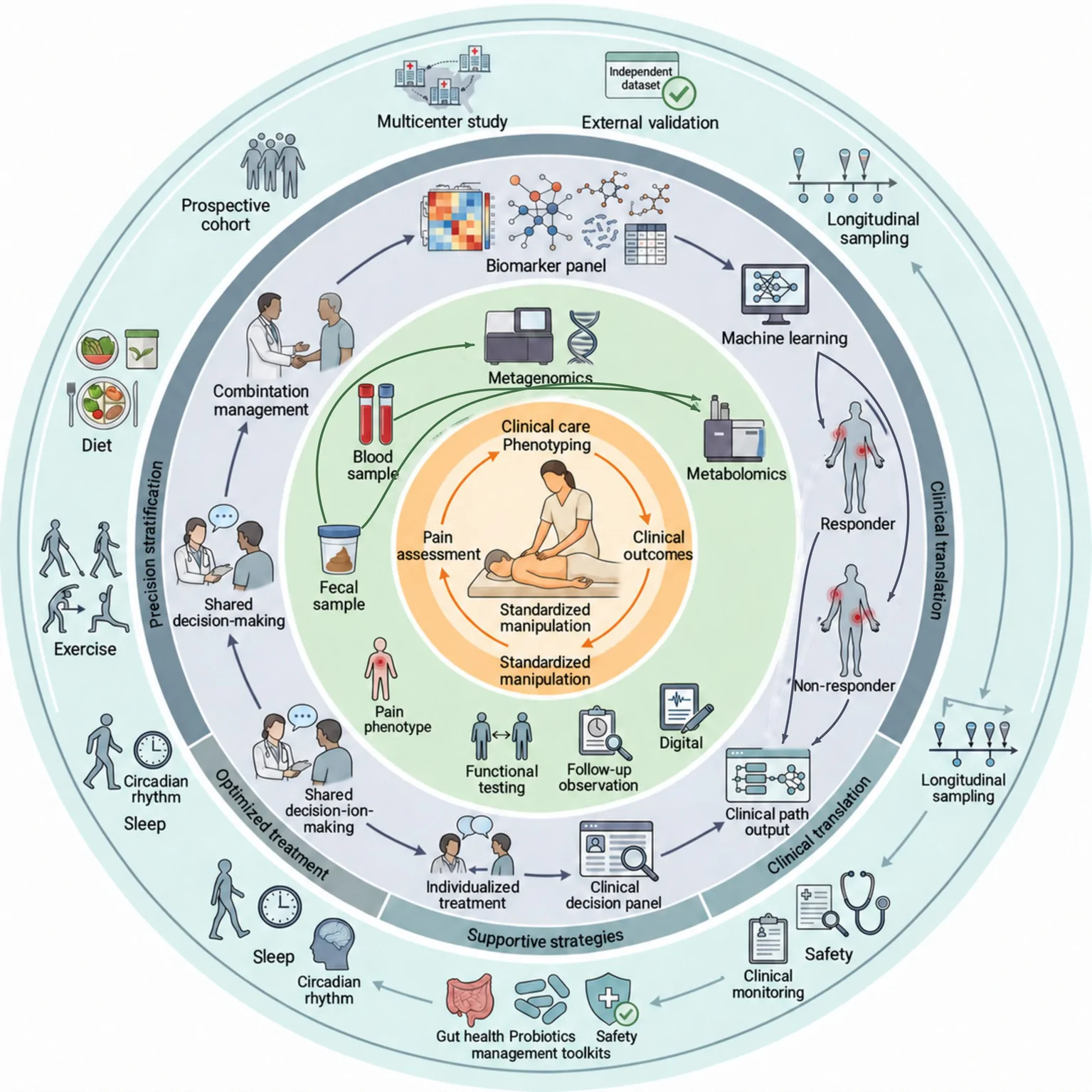
Standardization, therapeutic response stratification, and clinical translation pathway for multi-omics research on Tuina analgesia. The framework summarizes a research pathway from standardized Tuina intervention, clinical phenotyping, and biospecimen collection to metagenomic, metabolomic, and functional analyses, followed by candidate biomarker identification and therapeutic-response heterogeneity analysis. Longitudinal sampling, prospective cohorts, multicenter studies, and external validation are incorporated to assess stability and reproducibility, while diet, exercise, sleep, circadian rhythm, and safety are considered throughout the process. The framework outlines major steps for advancing exploratory multi-omics findings toward mechanistic and clinical validation.

### 7.5 Longitudinal observation, functional validation, and multicenter replication

Microecological and metabolic changes after Tuina may show marked temporal dynamics. Local mechanical responses, autonomic activity, inflammatory state, and tissue metabolism can change over different time scales after intervention, and comparisons restricted to before and after treatment cannot readily distinguish immediate stimulation responses, cumulative effects over a treatment course, and sustained changes after treatment has ended (Keter et al., 2025; Sun et al., 2026). Time-series multi-omics studies use repeated sampling to analyze dynamic relationships across biological layers, and related integration methods are increasingly being applied in microbiome research (Bodein et al., 2022; Sherwani et al., 2025). In Tuina research, simultaneous longitudinal measurement of mechanical stimulation dose, autonomic indices, host signals such as circulating myokines, microbial functions, metabolite profiles, and pain phenotypes during treatment and follow-up would help establish the temporal order of different changes and distinguish transient responses from more stable biological alterations.

Stable temporal associations can further narrow the candidate pathways requiring functional confirmation. When changes in microbial function show reproducible temporal relationships with corresponding metabolites, host signals, and pain phenotypes, targeted quantification and replication in independent samples can test their stability; microbiota transplantation, intervention with specific strains, metabolite supplementation, and receptor blockade can then assess the functional roles of key nodes (Metwaly et al., 2025; Gheorghe et al., 2021). Such functional studies have already provided specific examples in microbiome and pain research in which microbial communities or metabolites participate in pain phenotypes, but Tuina-related work still lacks continuous validation from mechanical stimulation to microbial function, metabolic signaling, and host targets. Concordance between longitudinal changes and functional experiments is therefore important for judging whether a multi-omics association has mechanistic significance.

The ability of findings to replicate across populations and research centers is further influenced by pain type, baseline patient status, practitioner, manipulation dose, sample handling, and omics platform. Manual therapy trials have documented insufficient intervention reporting and limited procedural reproducibility (Ruzich et al., 2024), while microbiome machine-learning studies also show that features identified in a single center or cohort may be substantially less stable in external cohorts (Li et al., 2023). Replication of the same candidate microbial function, metabolite, or treatment-response feature across treatment teams, regions, and analytical platforms therefore provides stronger evidence of stability than statistical significance in a single center. Standardization of Tuina intervention, longitudinal multi-omics observation, functional confirmation of key pathways, and cross-center replication together provide the basis for moving from associative findings toward more reliable mechanistic interpretation and clinical translation.

## 8 Conclusion

Chronic pain involves multilevel alterations in central sensitization, peripheral tissue status, neuroimmune responses, and metabolism, and gut microecology may participate in some of these cross-organ regulatory processes. Tuina-related studies have documented changes in local soft tissues, peripheral sensory input, autonomic activity, inflammation, and muscle metabolism, and a small number of studies have also reported changes in gut microbiota after intervention. However, studies continuously linking Tuina mechanical stimulation, gut microbial function, metabolic signaling, host receptors, and pain improvement remain limited. The brain, gut, and skeletal muscle may interact through autonomic pathways, immune-inflammatory signaling, circulating metabolites, myokines, and other distal signals, but many of these links are currently supported mainly by research in chronic pain, exercise physiology, and microbiome science.

Metagenomics can extend microbiota research from taxonomic composition to strains, functional genes, and metabolic pathways, while untargeted metabolomics captures metabolic changes jointly shaped by the host and microbiota. Together, these approaches can examine whether microbial functions such as SCFA production, bile acid transformation, and tryptophan metabolism change in parallel with measured metabolites, neuroimmune status, and pain phenotypes. Pain-mechanism studies provide experimental support for some links among microbial functions, metabolites, host receptors, and pain effects, but these pathways cannot be directly transferred as established mechanisms of Tuina analgesia. Metabolic changes after Tuina may also arise from local muscle responses, autonomic regulation, activity level, sleep, and emotional state; multi-omics associations therefore need to be interpreted in conjunction with temporal order, targeted quantification, and functional intervention.

The reliability of multi-omics research related to Tuina also depends on stable characterization of mechanical stimulation exposure, pain phenotype, sample collection, and analytical workflows. Quantification of manipulation parameters, longitudinal multi-time-point observation, control of key confounders, and cross-center replication can improve the interpretability of microecological and metabolic features, while functional studies involving microbiota transplantation, specific strains or metabolites, and receptor blockade can help determine whether key nodes actually participate in pain regulation. At present, microbiome and metabolome measures are used mainly to explain therapeutic-response heterogeneity and to identify biological features for further study; their value for response prediction and clinical decision-making remains insufficiently validated. Microecological-metabolic links across the brain, gut, and skeletal muscle therefore provide a testable cross-organ framework for Tuina analgesia, whose value will depend on whether future studies can connect stable omics associations with clearly defined functional mechanisms.
